# Defining T cell receptor repertoires using nanovial-based binding and functional screening

**DOI:** 10.1073/pnas.2320442121

**Published:** 2024-03-27

**Authors:** Doyeon Koo, Zhiyuan Mao, Robert Dimatteo, Miyako Noguchi, Natalie Tsubamoto, Jami McLaughlin, Wendy Tran, Sohyung Lee, Donghui Cheng, Joseph de Rutte, Giselle Burton Sojo, Owen N. Witte, Dino Di Carlo

**Affiliations:** ^a^Department of Bioengineering, University of California, Los Angeles, CA 90095; ^b^Department of Molecular and Medical Pharmacology, David Geffen School of Medicine, University of California, Los Angeles, CA 90095; ^c^Department of Chemical and Biomolecular Engineering, University of California, Los Angeles, CA 90095; ^d^Department of Microbiology, Immunology and Molecular Genetics, University of California, Los Angeles, CA 90095; ^e^Eli and Edythe Broad Center of Regenerative Medicine and Stem Cell Research, University of California, Los Angeles, CA 90095; ^f^Partillion Bioscience, Pasadena, CA 91107; ^g^Molecular Biology Institute, University of California, Los Angeles, CA 90095; ^h^Jonsson Comprehensive Cancer Center, University of California, Los Angeles, CA 90095; ^i^Parker Institute for Cancer Immunotherapy, David Geffen School of Medicine, University of California, Los Angeles, CA 90095; ^j^Department of Mechanical and Aerospace Engineering, University of California, Los Angeles, CA 90095; ^k^California NanoSystems Institute, Los Angeles, CA 90095

**Keywords:** TCR sequencing, single-cell secretion analysis, TCR immunotherapy

## Abstract

The ability to selectively bind to antigenic peptides and secrete effector molecules can define rare and low-affinity populations of cells with therapeutic potential in emerging T cell receptor (TCR) immunotherapies. We leverage cavity-containing hydrogel microparticles, called nanovials, each coated with peptide-major histocompatibility complex (pMHC) monomers to isolate antigen-reactive T cells. T cells are captured and activated by pMHCs inducing the secretion of effector molecules including IFN-γ and granzyme B that are accumulated on nanovials, allowing sorting based on both binding and function. The TCRs of sorted cells on nanovials are sequenced, recovering paired αβ-chains using microfluidic emulsion-based single-cell sequencing. By labeling nanovials having different pMHCs with unique oligonucleotide-barcodes and secretions with oligo-barcoded detection antibodies, we could accurately link TCR sequences to specific targets and rank each TCR based on the corresponding cell’s secretion level. Using the technique, we identified an expanded repertoire of functional TCRs targeting viral antigens with high specificity and found rare TCRs with activity against cancer-specific splicing-enhanced epitopes.

## Significance

T cells possess a vast diversity of surface receptors that bind to antigens presented on target cells, resulting in the activation of functions such as secretion of cytokines or cytotoxic molecules. T cell receptor (TCR) immunotherapies leverage this system to target tumor cells for elimination, yet methods of identifying rare TCRs remain nonspecific, resulting in many nonfunctional TCRs. We apply microcavity-containing hydrogel microparticles, known as nanovials, to selectively bind to and activate target T cells and capture secreted cytokines. This method enabled the linkage of TCR binding and functional secretion of cytokines directly with TCR sequences at the single-cell level, leading to expanded repertoires of TCRs and reduced false positives, ultimately enhancing the prospects of T cell cancer immunotherapy.

In the future, engineered cell therapies will be a pillar of medicine along with molecular and genetic interventions. There have been encouraging successes in the use of engineered T cell-based therapies, including T cell receptor (TCR) immunotherapy in treating cancer. These approaches use endogenous signaling activity in T cells and rely on the recognition of cancer-associated antigens that are presented as peptides associated with major histocompatibility complex (MHC) on the surface of tumor cells (1). Engineered TCRs have demonstrated efficacy in treating multiple types of tumors including melanoma, sarcoma, and leukemia (2, 3).

One technical hurdle for developing effective TCR immunotherapy is to identify reactive TCRs that can recognize targets with sufficient affinity and potency. T cells have one of the most diverse sequence repertoires (10^8^ to 10^20^) to respond to a wide variety of pathogens (4, 5). Current tools for enriching and screening cognate T cell populations rely mostly on TCR affinity or function, as defined by surface or intracellular markers of lymphocyte activation. Peptide-MHC (pMHC) multimer (e.g., tetramer) staining is the conventional method to specifically label T cells with cognate TCRs benefiting from the avidity effect when four pMHC monomers are linked through a tetrameric streptavidin backbone (6). However, pMHC multimer staining does not take into account the functional stages of the T cells, and binding of multimers to T cells is not always correlated with activation or cytotoxicity (6).

An alternative way to isolate reactive T cells is through both extracellular and intracellular activation markers (7–9). The stimulation of T cells based on activation biomarkers can be achieved without the knowledge of specific epitopes and the readouts of these markers are based largely on functional activation of the T cell (7, 8). Despite improvement in these activation-based selection techniques and better choices of markers, some of the T cells isolated by surface markers have been reported to be “bystander T cells,” meaning that they were not able to respond to antigens in a reconstructed experiment (8). Techniques based on intracellular markers, on the other hand, require cell fixation and permeabilization leading to less RNA recovery at lower quality and may still recover TCRs that are noncytolytic, as noncytotoxic cells can secrete interferons (IFNs) and tumor necrosis factors (TNFs) (10, 11). For example, our previous attempts to recover TCRs targeting cancer-enhanced splicing variants identified 8 functional TCRs from 389 candidate TCR sequences in total recovered by either surface (CD137) or intracellular (IFNγ and TNFα) markers (12). Secreted granzyme B is thought to be a specific marker for epitope-induced cytotoxic cells, but traditional methods like FACS and ELISPOT are not compatible with sorting of live single cells (13, 14). An ideal technology would combine antigen-specific enrichment and secretion-based screening to achieve both highly specific identification of functional TCR sequences along with the knowledge of their cognate target epitopes.

**Fig. 1.**
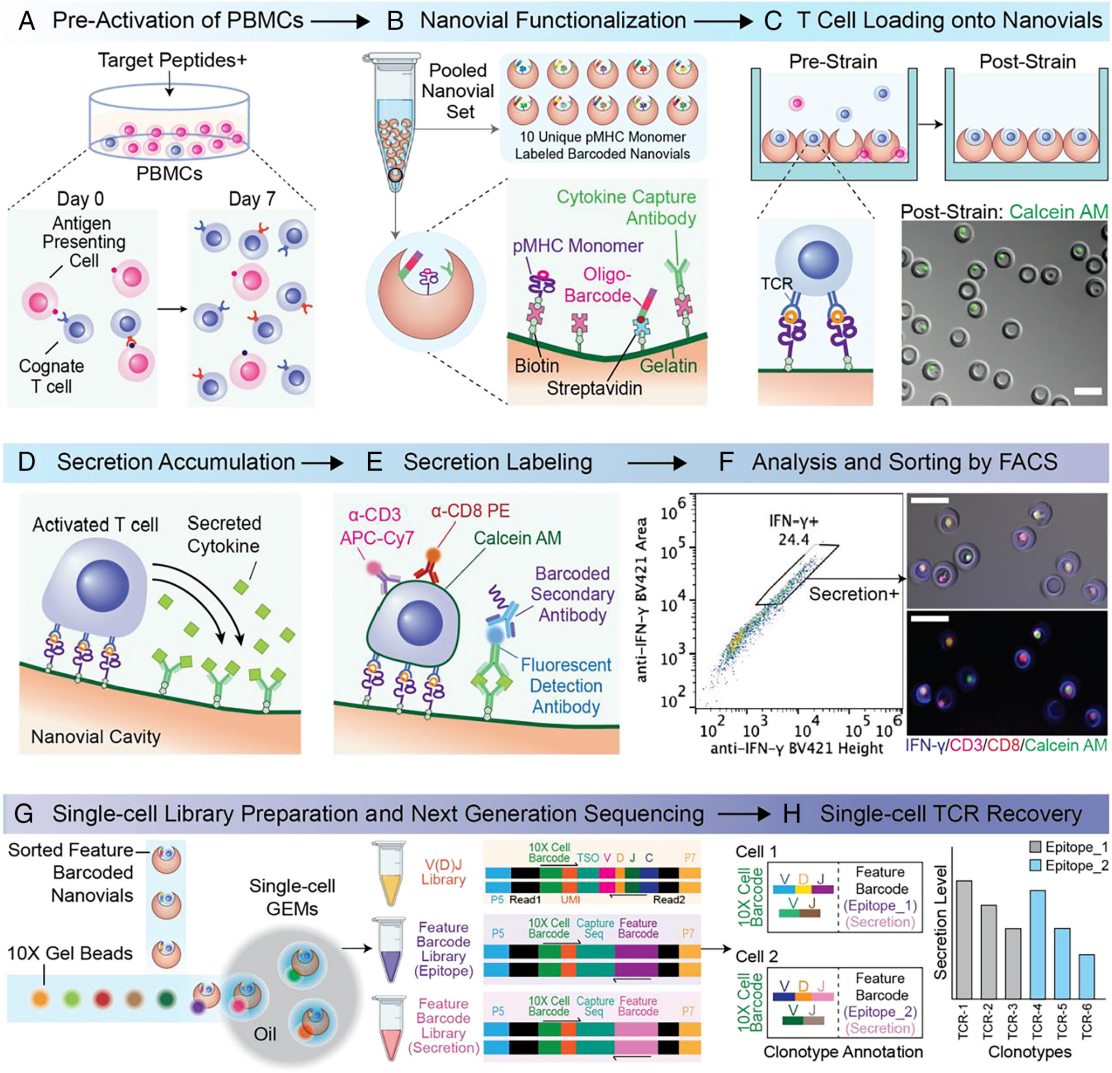
Overview of high-throughput analysis and isolation of antigen-specific T cells followed by recovery of a single-cell TCR library. (*A*) Optional pre-expansion of PBMCs with target peptides for 7 d. (*B*) Functionalization of nanovials with secretion capture antibodies, pMHC monomers, and oligonucleotide barcodes via streptavidin-biotin chemistry. (*C*) Loading of cognate T cells into the cavities of nanovials in a well plate and removal of unbound cells using a cell strainer. (*D*) Activation of T cells for 3 h and secretion capture in the cavity of nanovials. (*E*) Labeling of captured cytokines and cell surface markers with fluorescent detection antibodies, followed by oligonucleotide barcoded antibodies against secreted markers. (*F*) Sorting of cells on nanovials based on viability, CD3/CD8 expression, and secretion signal. (*G*) Compartmentalization of sorted population into droplets with a cell barcode bead in the 10X Chromium system for the construction of matched V(D)J and feature barcode libraries (nanovial-epitope and secretion barcodes). (*H*) Annotation of TCR clonotypes with corresponding secretion levels and epitopes by matching feature barcodes. (Scale bars represent 50 μm.)

We recently reported an approach to confine cells in small nanoliter-volume cavities within hydrogel microparticles, which we call “nanovials,” and capture secreted molecules on the nanovial surfaces (15, 16). Here, we adapted the nanovial technology to achieve combined antigen-specific capture and functional activation-based high-throughput analysis and sorting of live single T cells based on secreted cytokines (Fig. 1). Each nanovial acts as both an artificial antigen-presenting cell that presents pMHC molecules at high valency within the cavity to capture with high avidity and activate cells with cognate TCRs, and as a capture site for secreted molecules, allowing accurate measurement of secreted effector molecules, such as granzyme B.

To recover TCR sequences in an epitope-specific manner, live cells on nanovials are sorted based on CD3 and CD8 expression and secretion (i.e., IFN-γ, granzyme B), followed by single-cell sequencing to construct a single-cell TCR library with matching αβ-chains. Corresponding antigen-specific information and secretion amount are linked to each TCR sequence using oligonucleotide feature barcodes encoding the specific pMHC molecules on the nanovial and an antibody targeting the secretion detection antibody (Fig. 1 *B* and *E*). Using this platform, we were able to find an expanded number of viral-epitope-specific cognate TCRs compared to tetramers and recover rare prostate cancer-specific functional TCRs that emerged as promising candidates when linking single-cell secretion to each TCR sequence.

## Results

### Fabrication and Functionalization of Nanovials for T Cells.

Our first aim was to functionalize nanovials to capture T cells and secreted cytokines. We used a microfluidic device that generates uniform water-in-oil emulsions to create millions of monodisperse polyethylene glycol (PEG)-based nanovials with an inner cavity selectively coated with biotinylated gelatin (*SI Appendix*, Fig. S1*A*) (15, 16). To accommodate human T cells with diameters of ~10 μm, we fabricated uniform nanovials with an average outer diameter of 35 μm (CV = 5.1%) and an average cavity diameter of 21.2 μm (CV = 7.2%). By functionalizing the inner cavity with biotin during fabrication, we could flexibly link multiple biotinylated antibodies or peptide-MHC (pMHC) monomers with epitopes of interest through streptavidin-biotin noncovalent interactions (Fig. 1*B*). For capture and secretion analysis of primary human T cells, irrespective of antigen targeting, we decorated nanovials with biotinylated anti-CD45 and cytokine capture antibodies against interferon-γ, tumor necrosis factor-α, and interleukin-2 (anti-IFN-γ, anti-TNF-α, and anti-IL 2). At least a two order of magnitude dynamic range in detection of recombinant cytokines was observed when anti-CD45 capture antibodies and one, 1:1 (140 nM each) or two cytokine secretion capture antibodies, 1:1:1 (140 nM each) were used during functionalization (*SI Appendix*, Fig. S1*B*). All of these conditions allowed for T cell loading (*SI Appendix*, Fig. S2) as well as signal capture from recombinant cytokines down to 10 ng/mL (*SI Appendix*, Fig. S1*B*). Cell loading followed Poisson loading statistics with an optimum observed when 1.6 cells per nanovial were seeded (*SI Appendix*, Fig. S2*A*) with anti-CD45 used to capture cells (*SI Appendix*, Fig. S2*B*) and was largely independent of the presence of additional cytokine capture antibodies (*SI Appendix*, Fig. S2*C*). To capture antigen-specific T cells, nanovials were decorated with pMHC monomer along with cytokine capture antibodies. We saw a linear increase in loaded pMHC monomer signal up to concentrations of 80 µg/mL, resulting in nanovials that could act as artificial antigen presenting cell with high valency of pMHC monomers (*SI Appendix*, Fig. S1*C*). In order to maintain sites for capture antibodies targeting secreted cytokines, we limited the pMHC concentration to 20 µg/mL for future experiments unless otherwise stated.

Having validated recombinant cytokine assays and T cell loading on nanovials, we wanted to test the processes to accumulate and detect secretions from single T cells bound to nanovials using flow cytometric analysis. We developed assays for three secreted cytokines (ΙFN-γ, ΤΝF-α, and IL-2) using nanovials coated with the respective individual cytokine capture antibody and anti-CD45. After loading primary human T cells onto nanovials, cells were activated nonspecifically with phorbol 12-myristate 13-acetate (PMA) and ionomycin for 3 h. Following fluorescent staining of captured cytokines, we used a standard cell sorter, the SONY SH800S, to sort nanovials with each cytokine secretion signal based on fluorescence peak area and height values (*SI Appendix*, Note S1). By also gating on a cell viability dye, such as calcein AM, we were able to simultaneously measure secretions and viability of individual cells on nanovials, improving the selective sorting of functional cells (*SI Appendix*, Fig. S3*A*). We gated and sorted populations into low, medium, or high secretors based on the area vs. height plot, recovering viable cells with different levels of TNF-α and IFN-γ secretion as reflected in fluorescence microscopy images (*SI Appendix*, Fig. S3*B*). We also evaluated crosstalk between nanovials by coculturing cell-loaded nanovials and fluorescently labeled (AlexaFluor 488) nanovials without cells and found that less than 0.04% of test nanovials without cells appeared in the positive secretion gate during the 3 h activation period (*SI Appendix*, Fig. S3*C*). Presumably, secreted cytokines accumulate at higher concentrations locally but when cytokines diffuse or are advected to neighboring cavities, the concentration is diluted substantially leading to a reduced signal.

### Capture of Antigen-reactive T Cells with pMHC-labeled Nanovials.

**Fig. 2.**
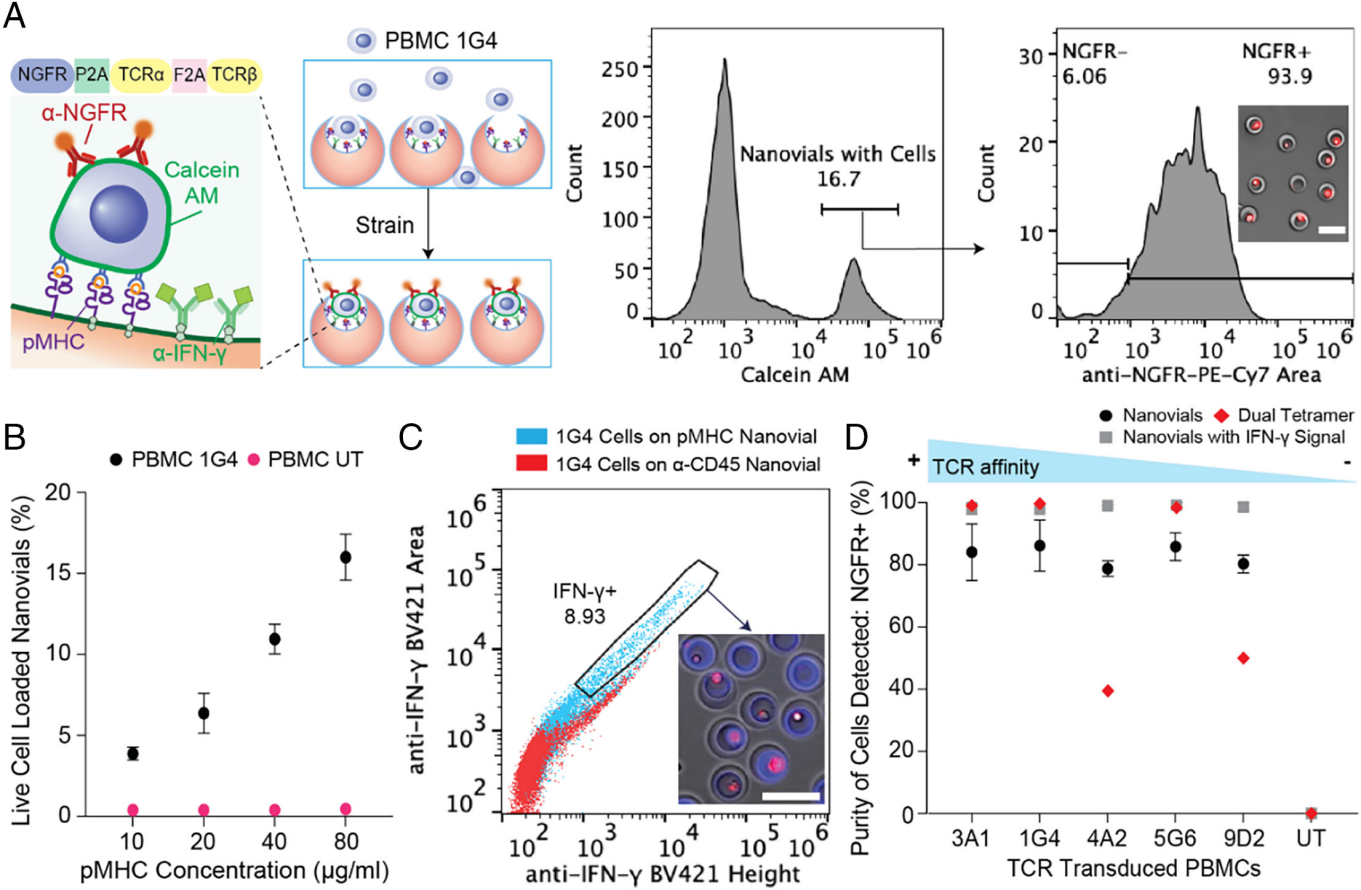
Detection of antigen-specific T cells on HLA-A*02:01 restricted NY-ESO-1 pMHC labeled nanovials. (*A*) PBMCs transduced with 1G4 TCRs are captured onto NY-ESO-1 pMHC labeled nanovials with ~94% of bound cells staining positive for anti-NGFR. (Scale bar represents 50 μm.) (*B*) The fraction of nanovials containing live cells plotted as a function of pMHC concentration for 1G4-transduced (black dots) or untransduced PBMCs (magenta dots). (*C*) Flow cytometry plots of IFN-γ secretion induced by nanovials at 3 h for 1G4-transduced PBMCs loaded onto anti-CD45 labeled nanovials (red dots) or NY-ESO-1 pMHC labeled nanovials (cyan dots). Secreting cells on pMHC-labeled nanovials sorted from the gated area are shown. (Scale bar represents 50 μm.) (*D*) The purity of recovered cognate T cells with various affinities to HLA-A*02:01 restricted NY-ESO-1 pMHC are shown. Measurements are based on binding to nanovials (black circles), nanovials with IFN-γ secretion (grey squares), or labeling with dual-color tetramers (red diamonds) as a function of TCR.

We hypothesized that nanovials coated with pMHC and cytokine capture antibodies could be used for antigen-specific capture, TCR-specific activation, and detection of secreting cytokines. Transitioning from using anti-CD45, pMHC-functionalized nanovials were applied for the selection of antigen-reactive T cells. We first analyzed the specificity of nanovials in selectively binding antigen-specific T cells using human peripheral blood mononuclear cells (PBMCs) transduced with 1G4 TCR targeting NY-ESO-1, a clinically studied cancer-specific antigen (3, 17). Truncated nerve growth factor receptor (NGFR) was used as the cotransduction marker for the presence of 1G4 TCR. PBMCs transduced with and expressing 1G4 bound specifically to nanovials labeled with pMHC monomer containing HLA-A*02:01 restricted NY-ESO-1 C9V peptide (SLLMWITQV) (20 µg/mL) (Fig. 2*A*). Live cells occupied ~17% of nanovials, and 93.9% had NGFR expression. To further clarify whether the interaction was specific, we increased the NY-ESO-1 pMHC concentration used to functionalize nanovials. A corresponding increase in the amount of antigen-specific T cells loaded was observed in a dose-dependent manner, while the binding of untransduced PBMCs was low and independent of pMHC concentration (Fig. 2*B* and *SI Appendix*, Fig. S4*A*).

To investigate whether pMHCs on nanovials can specifically trigger activation and secretion from engaged antigen-specific T cells, 1G4-transduced cells were each loaded onto nanovials labeled with pMHC monomers or anti-CD45 antibodies. Cells loaded on control anti-CD45 labeled nanovials (red dots) had low levels of IFN-γ signal, which was mostly associated with nonspecific staining of cells, while cells on NY-ESO-1 pMHC-labeled nanovials (cyan dots) clearly secreted IFN-γ as early as 3 h after loading (*SI Appendix*, Fig. S4*B*), yielding one to two orders of magnitude higher fluorescence intensity observed at larger area:height ratios (Fig. 2*C*).

For some therapeutic workflows, enrichment and regrowth of rare antigen-reactive populations are required. To assess proliferation of antigen-specific T cells after isolation, 1G4-transduced PBMCs enriched on NY-ESO-1 pMHC-coated nanovials were first sorted and detached using collagenase D. Cells expanded in culture over 5 d, with 90% of the expanded population expressing the 1G4 TCR, showing enrichment and continued growth of the antigen-specific T cell population (*SI Appendix*, Fig. S4*C*).

### T Cells with Low-affinity TCRs Are Isolated Effectively by pMHC-coated Nanovials.

Since the 1G4 TCR has high affinity to NY-ESO-1 pMHC, we questioned whether increased avidity of pMHCs coating the nanovial cavity would prove advantageous in recovering TCRs with various affinities. Human PBMCs were transduced with five previously identified TCRs (3A1, 1G4, 4A2, 5G6, 9D2) targeting the same HLA-A*02:01 restricted NY-ESO-1 C9V peptide. The relative affinities of these TCRs were assessed using fluorescent MHC dextramer binding (17). We compared the purity of antigen-specific cells by nanovial capture, nanovial capture gated on IFN-γ secretion, and dual-color tetramer staining (Fig. 2*D*). The purity of detected cells was defined as the fraction of NGFR^+^ cells from CD3^+^/CD8^+^ cells on nanovials with or without IFN-γ secretion signal, or fraction of NGFR^+^ cells from dual-tetramer^+^ cells (*SI Appendix*, Fig. S5 *A* and *B*). The purity of isolated cells on nanovials approaches 100% for NGFR+ cells when also considering IFN-γ signal (Fig. 2*D*). pMHC-labeled nanovials also recovered more antigen-specific T cells than dual-color tetramer, especially when cells possessed low-affinity TCRs (4A2, 5G6, 9D2) as represented by the total NGFR+ cells in *SI Appendix*, Fig. S5*C*.

### Recovery of Functional Viral-epitope-specific TCRs using Nanovials.

**Fig. 3.**
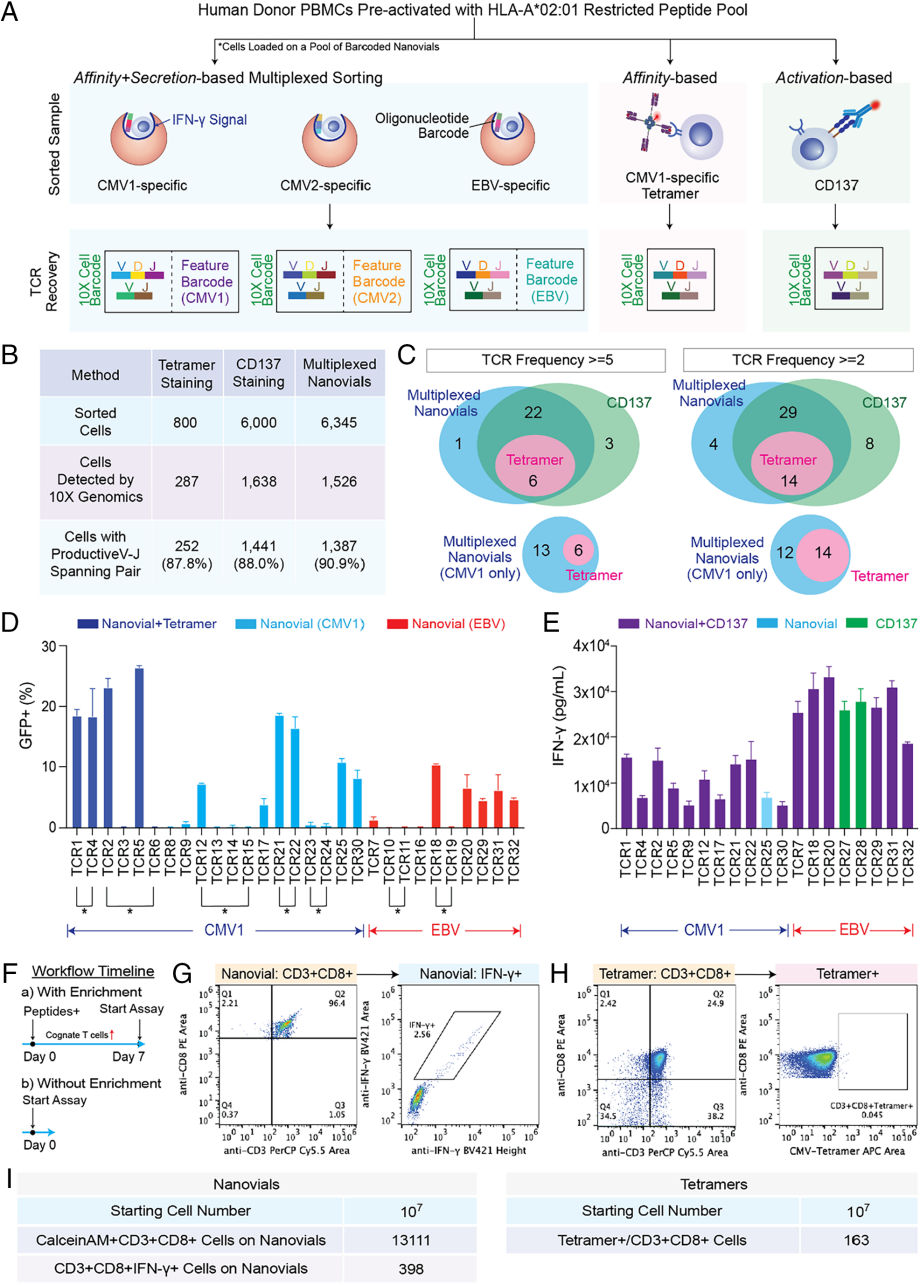
Sorting of antigen-specific cells and recovery of single-cell TCR clonotypes using nanovial, tetramer, or CD137 approaches. (*A*) TCR identification workflow and matching epitope deconvolution using nanovials along with comparison techniques. For nanovials, each TCR was matched with a corresponding oligonucleotide barcode sequence reflecting pMHC information. (*B*) Summary of single-cell TCR αβ sequencing results. Sorted cells refers to the number of cells gated and sorted as positive (see *SI Appendix*, Fig. S6 for detailed gates). (*C*) A representative Venn diagram of recovered αβ-paired TCR sequences from three approaches with a frequency ≥5 (*Left*) or ≥2 (*Right*). (*D*) The percent of GFP+/CD8+/murineTCRβ+ cells from NFAT-GFP reporter Jurkat cells transduced with respective TCR and exposed to APCs with exogenously added peptides. CMV1-reactive TCRs recovered by both nanovial and tetramer approaches (dark blue) and only nanovials (light blue) or EBV-reactive TCRs recovered by nanovials (red) are plotted. Connecting lines and an asterisk represent TCRs recovered from the same cell clonotype. Venn diagram showing four overlapping and six additional TCRs recovered by nanovials compared to CMV1 pMHC tetramers. (*E*) IFN-γ secretion measured by ELISA is plotted following exposure of PBMCs transduced with the same 19 reactive TCRs to APCs with added peptide. No secretion was observed from the negative control group, PBMCs transduced with the same vector but without TCRs. TCRs enriched with CDR3s for the same motif are represented by a connecting line and motif nomenclature. (*F*) Workflow representing direct ex vivo analysis without a preactivation expansion and enrichment process. (*G*) Flow cytometry plots of freshly thawed PBMCs showing gates for CD3^+^CD8^+^ cells and IFN-γ secretion signal using nanovials coated with CMV1-specific pMHC. (*H*) Flow cytometry plots of the same PBMC population using tetramer staining. Gates for CD3^+^CD8^+^ cells and tetramer positivity are shown. (*I*) Summary of rare CMV1-specific T cells isolated by nanovial or tetramer methods.

Following successful isolation of rare antigen-specific T cells with TCRs of varying affinities in model systems we hypothesized that sorting based on a combination of binding and cytokine secretion using nanovials would increase the functional hit rate of a diverse repertoire of TCRs specific to common viral epitopes. Healthy donor PBMCs preactivated with a pool of previously reported HLA-A*02:01 restricted peptides from cytomegalovirus (CMV) and Epstein Barr virus (EBV) targeting CMV pp65 (CMV1, NLVPMVATV), CMV IE-1 (CMV2, VLEETSVML), and EBV BMLF1 (EBV, GLCTLVAML) were isolated using three different methods: secretion based sorting using nanovials, sorting using a CMV pp65-specific tetramer, or activation-based sorting using CD137 as the surface marker (Fig. 3*A*). We multiplexed the detection of antigen-specific T cells by loading cells onto a pool of barcoded nanovials labeled with three HLA-A*02:01 restricted pMHCs targeting each antigen (CMV1, CMV2, and EBV) and a corresponding oligonucleotide barcode. Approximately 6,000 CD3^+^CD8^+^ cells on nanovials associated with IFN-γ secretion signal and 800 CMV1-specific pMHC tetramer+ cells were identified and sorted from the entire sample (Fig. 3*B* and *SI Appendix*, Fig. S6). To have an equivalent starting cell number for single-cell sequencing, 6,000 cells were sorted based on gating for above background levels of the CD137 activation marker (*SI Appendix*, Fig. S6).

TCRs were recovered from sorted cells using the 10X Genomics Chromium platform (Fig. 3*B*). Notably, the cells on nanovials were introduced directly into the system to maintain the connection between a nanovial with a feature barcode oligonucleotide tag and the attached T cell. Nanovials did not interfere with the gene sequence recovery resulting in the highest fraction of cells with a productive V–J spanning pair (90.9%) compared to tetramer (87.8%) and CD137 samples (88%) (Fig. 3*B*). We compiled a list of high-frequency TCR clonotypes (frequency ≥ 5) detected by the three methods. Since a few clonotypes contained multiple alpha or beta chains, we recombined them into separate TCR sequences with each permutation of alpha and beta chains. In total, we retrieved 32 unique TCR pairs with frequency ≥5: 6 TCRs overlapped among the three methods, 28 overlapped between the nanovial and CD137 approaches, and one unique TCR was detected with nanovials (Fig. 3*C*). A larger number of unique TCR sequences were detected with a less stringent cutoff of frequency ≥2, where the overlapping number of TCRs between nanovial and CD137 techniques increased from 28 to 43, suggesting additional rarer TCRs were also found.

### Barcoded Nanovials Reveal Epitope Information during T Cell Isolation.

Unlike workflows using CD137, which require laborious deconvolution to uncover the target epitopes from a peptide pool that match specific TCR sequences, pMHC-barcoded and multiplexed nanovials reveal epitope information during cognate T cell isolation. Using the 10X Chromium system, TCR sequence information of each cell was linked to the nanovial pMHC feature barcode, resulting in the recovery of each TCR with matching target epitope information. A >90% frequency of the pMHC barcode identified the dominant epitope for each TCR (*SI Appendix*, Fig. S7). The distribution of clonotype frequency with the corresponding epitope is represented in *SI Appendix*, Fig. S6*C* for the nanovial workflow. We detected 32 CMV1 (CMV pp65), 1 CMV2 (CMV IE1), and 12 EBV-reactive clonotypes, and 96.4% of sequenced cells with productive V(D)J spanning pairs had matching epitope information.

To understand the antigen-specific reactivity of 32 unique TCR sequences from 26 clonotypes (one clonotype may contain multiple alpha and beta chains) retrieved by the three methods (nanovial, tetramer, CD137) with a frequency ≥5, candidates were re-expressed via electroporation into Jurkat-NFAT-GFP cells, in which GFP expression can be induced upon TCR recognition. Murine constant regions were used for both TCR alpha and beta chains to prevent mispairing with endogenous TCRs. Engineered Jurkat cells were then cocultured with K562 cells expressing HLA-A*02:01 (K562-A2) as antigen-presenting cells along with exogenously added peptides. Activation of the Jurkat cells was determined by flow cytometry, gating on % of the CD8^+^/murineTCRβ^+^ population with GFP signal above background. K562 cells with exogenously added DMSO (instead of cognate peptide) were used as a control to measure nonspecific activation of each TCR (*SI Appendix*, Fig. S11). From the smaller pool of CMV1-specific TCRs, the nanovial workflow yielded six more reactive TCRs compared to CMV1 pMHC tetramer labeling (Fig. 3*D*). Out of the 29 possible TCR combinations identified with nanovials, 17 were found to be reactive upon re-expression (11 for CMV1 and 6 for EBV) (Fig. 3*D*). Notably, some of these TCRs were from cells in which sequencing yielded multiple alpha and/or beta chains (denoted with connecting line and an asterisk, Fig. 3*D*). When collapsing these related TCR clonotypes to individual cell clonotypes, we found that 78% of clonotypes recovered using nanovials had at least one TCR permutation with antigen-specific reactivity and the calling of epitope information was accurate for those reactive TCRs. The few nonreactive TCRs were tested with the other peptides and found to be unreactive.

To investigate how functional IFN-γ secretion-based selection on nanovials correlated to secretory function elicited by the recovered TCR sequences in T cells, 19 reactive TCRs identified in the Jurkat-NFAT-GFP assay were transduced into human PBMCs and IFN-γ secretion was measured following exposure to antigen-presenting cells (APCs) with exogenously added cognate peptides. We found that T cells transduced with all 19 reactive TCRs tested were able to specifically produce secreted IFN-γ (>5,000 pg/mL) when stimulated by APCs presenting exogenous peptides (Fig. 3*E*). Levels of secreted IFN-γ in PBMCs were not directly correlated to GFP activation signals when tested in Jurkat-NFAT-GFP (R^2^ = 0.10). Nanovial and CD137 approaches were both able to recover TCRs with a range of different potencies, but only nanovials provided matched epitope information.

### Direct Enrichment of Antigen-specific T Cells on Nanovials and Comparison to Tetramers.

Since a preactivation expansion step of PBMCs was used to enrich reactive T cells (Fig. 3*F*), requiring an additional 7 d of culture, we asked whether nanovials could directly enrich and activate T cells from freshly thawed PBMCs. We loaded PBMCs directly onto nanovials or performed tetramer staining, both using pMHC CMV1 (CMV pp65, NLVPMVATV). Then, 10^7^ PBMCs were used in each method without pre-expansion, which reduces a week-long experiment to a single day (Fig. 3*F*). Antigen-specific T cells that bound to CMV1 on nanovials and secreted IFN-γ or bound to CMV1 on tetramers were gated and recovered by the two methods (Fig. 3 *G* and *H*). pMHC-labeled nanovials were able to recover ~13,000 CD3^+^CD8^+^ cells with a clear fraction of bound cells (398) secreting IFN-γ (Fig. 3*I*). For the same sample of PBMCs, using duo-color tetramers yielded 163 CD3+CD8+ cells (Fig. 3*I*).

### Detection of Antigen-specific T Cells based on Granzyme B Secretion using Nanovials.

**Fig. 4.**
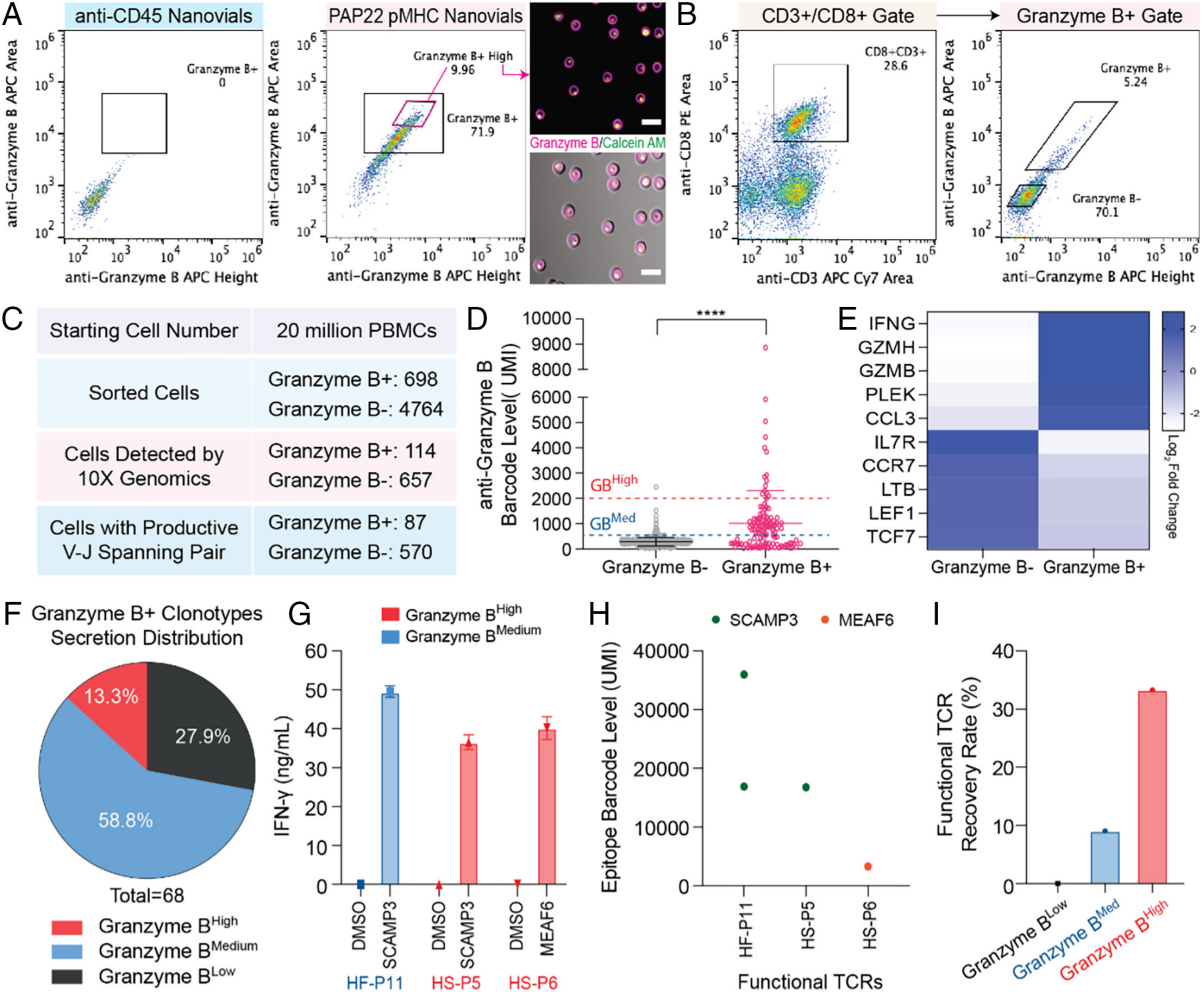
Identification of rare functional TCRs using granzyme B secretion-based nanovial assay. (*A*) Flow cytometry plots of granzyme B secretion specifically induced by pMHC-labeled nanovials at 3 h for TCR156 transduced PBMCs loaded onto anti-CD45 labeled nanovials or PAP22 pMHC labeled nanovials. Secreting cells on pMHC-labeled nanovials sorted from the gated area are shown. (Scale bars represent 50 μm.) (*B*) Flow cytometry plots of human donor PBMCs loaded onto nanovials and secreting granzyme B. CD3+CD8+ cells on nanovials were sorted as granzyme B secreting (Granzyme B+) or nonsecreting cells (Granzyme B−). (*C*) Summary of single-cell TCR αβ sequencing results. Sorted cells refer to the number of cells gated and sorted as positive or negative. (*D*) Anti-granzyme B barcode levels for Granzyme B+ and Granzyme B− populations. Dashed lines represent the secretion barcode level cut-off: red Granzyme BHigh (barcode > 2,000), blue Granzyme BMedium (2,000 > barcode > 500). (*E*) Top 10 differentially expressed genes among Granzyme B+ and Granzyme B− populations. (*F*) Secretion phenotype distribution of Granzyme B+ clonotypes classified as Granzyme BHigh, Granzyme BMedium, and Granzyme BLow. (*G*) Identification of three functional TCRs validated upon re-expression in human PBMCs and measurement of IFN-γ secretion following exposure to APCs with added peptides. IFN-γ secretion measurement of all 25 TCRs tested are represented in *SI Appendix*, Fig. S8*B*. Two TCRs from the Granzyme BHigh (red bar) and one TCR from Granzyme BMedium (blue bar) populations were found to be functional. (*H*) Nanovials accurately recovered the epitope information encoding specific pMHC molecules for the three functional TCRs in *G*. (*I*) The highest recovery rate of functional TCRs was observed from TCRs recovered based on the highest granzyme B secretion barcode signal.

IFN-γ signaling is primarily associated with activated T cells and cell-mediated immune responses (18). As more direct evidence for cytotoxicity of antigen-specific T cells, we further expanded the nanovial assay for the isolation of T cells based on granzyme B secretion, which remains challenging by currently available techniques (11, 13, 14). A previously identified TCR (TCR156) targeting a defined epitope (PAP22) of prostatic acid phosphatase (PAP), a prostate tissue antigen, was used to validate this approach (19). This low-affinity TCR shows antigen-specific recognition but weak tetramer signals in reconstruction experiments (19). In the context of HLA-A*02:01, TCR156-transduced PBMCs were loaded onto anti-CD45-labeled or PAP22 pMHC-labeled nanovials, and granzyme B secretion was analyzed after 3 h of activation. Strong granzyme B secretion was only observed from the cells that bound to pMHC-labeled nanovials, showing antigen-specific activation (Fig. 4*A*). By sorting the top 10% of granzyme B secreting cells, we confirmed viability and intense secretion signal on the nanovial cavity by fluorescence microscopy (Fig. 4*A*).

### Finding Rare Functional TCRs Targeting Prostate Cancer Epitopes.

We then wanted to challenge our nanovial platform for the recovery of rare functional TCRs targeting PAP and cancer-enhanced splicing peptides from human donor PBMCs. Previous studies indicate the frequency of finding cognate TCRs against those epitopes is extremely low (12, 19). In this experiment, we expanded the number of nanovial types to 10 different HLA-A*02:01 restricted pMHC-labeled barcoded sets: PAP14 (ILLWQPIPV), PAP21 (LLLARAASLSL), PAP22 (TLMSAMTNL), PAP23 (LLFFWLDRSVLA), CTNND1 (MQDEGQESL), CLASP1 (SLDGTTTKA), MEAF6 (SGMFDYDFEYV), PXDN (HLFDSVFRFL), SCAMP3 (STMYYLWML), and TCF12 (SLHSLKNRV), all of which have been previously used for TCR finding (12, 19). In order to increase the confidence in re-expressing potential rare TCRs with low frequency of recovery, we also introduced a unique capability into the nanovial assay where we link cell secretion of granzyme B to the TCR sequence information by adding an oligo-nucleotide barcoded antibody that reports out the level of granzyme B secretion. In this case, an oligo-anti-APC antibody targeting anti-granzyme B-APC was added. We aimed to be able to rank TCR sequences by the amount of granzyme B associated with T cells expressing that TCR. Starting with 20 million donor PBMCs from one healthy donor, live+CD3+CD8+ cells that bound to nanovials and had granzyme B signal above the gate (granzyme B+, 698 cells) were sorted (Fig. 4*B*). A subset of live+CD3+CD8+ cells on nanovials below the granzyme B secretion threshold (granzyme B−, 4764 cells) were also sorted as a negative control (Fig. 4*B*). Using the 10X Genomics platform, we constructed libraries for V(D)J sequences, the 1st feature barcode encoding the specific pMHC molecule (of 10 types) on nanovial, the 2nd feature barcode encoding granzyme B secretion level, and gene expression. In total, we sequenced and recovered 87 cells with a productive V–J spanning pair from the Granzyme B+ population and 570 cells from the Granzyme B− population (Fig. 4*C*).

Using the oligo-barcoded detection antibodies targeting the granzyme B signal, we were able to identify the secretion level for each cell (Fig. 4*D*). As expected, the average secretion barcode level of the Granzyme B+ population (Mean = 1,022, SD = 1,286) was significantly higher (*P* < 0.0001) than the Granzyme B- population (Mean = 288, SD = 149), representing that the oligo-barcoding process accurately reflects the fluorescence gates (Fig. 4*D*). Notably, the three most differentially expressed up-regulated genes among the Granzyme B+ population as compared to the Granzyme B− population were IFN-γ, granzyme H, and granzyme B, which supports the idea that granzyme B and IFN-γ act as crucial effectors for inducing cytotoxic activity (Fig. 4*E*) (20–22).

Based on the distribution of granzyme B secretion barcode levels, we categorized each T cell subset into three different classes: Granzyme B^High^ (barcode level ≥ 2,000), Granzyme B^Medium^ (2,000 > barcode level ≥ 500), Granzyme B^Low^ (barcode level < 500) (Fig. 4*D*). Among the 68 TCR candidates that belong to the Granzyme B+ class, 40 fell under Granzyme B^Medium^ (58.8%), 9 under Graznyme B^High^ (13.3%) and 19 under Granzyme B^Low^ (27.9%) (Fig. 4*F*).

### Functional Validation of TCRs Recovered based on Secretion Barcode Levels.

We hypothesized that T cells with the highest levels of granzyme B would yield the most potent TCRs with the highest reactivity. We ranked and selected the top six clonotypes from the Granzyme B+ population expressing granzyme B secretion barcode levels above 2,000 with productive TCR alpha and beta chains (HS-P1 to HS-P6) (*SI Appendix*, Fig. S8*A*). In comparison, we also tested the 15 most frequent clonotypes, following common practice for identifying potent TCRs (HF-P1 to HF-P15). Interestingly, no clonotypes in the high-frequency list overlapped with the Granzyme B^high^ clonotypes (*SI Appendix*, Fig. S8*A*).

To evaluate the functionality of recovered clonotypes in these separate lists, we re-expressed each candidate in human PBMCs and IFN-γ secretion was measured following exposure to antigen-presenting cells (APCs) with exogenously added cognate peptides (peptide pool or a single-peptide noted from the nanovial barcode encoding the specific pMHC molecule). A few clonotypes containing αβ chain permutation were recombined into a separate TCR sequence with each permutation of αβ chains. In total, we validated 25 unique TCRs: 19 TCRs recovered based on high frequency and six TCRs recovered based on high secretion (*SI Appendix*, Fig. S8*B*). From PBMCs of one healthy donor, we found two functional TCRs from the Granzyme B^High^ and one from Granzyme B^Medium^ TCRs that secreted IFN-γ upon exposure to APCs (Fig. 4*G*). For those 3 functional TCRs, nanovials also provided accurate epitope information (Fig. 4*H*). The recovery rate of functional TCRs from Granzyme B^High^ was highest (33%) as compared to Granzyme B^Medium^ (6.7%) and Granzyme B^Low^ (0%) TCRs, suggesting that functional (secretion) information captured by nanovials improves the detection rate of rare and potent TCRs (Fig. 4*I*).

### Multiplexed Secretion-based Profiling.

**Fig. 5.**
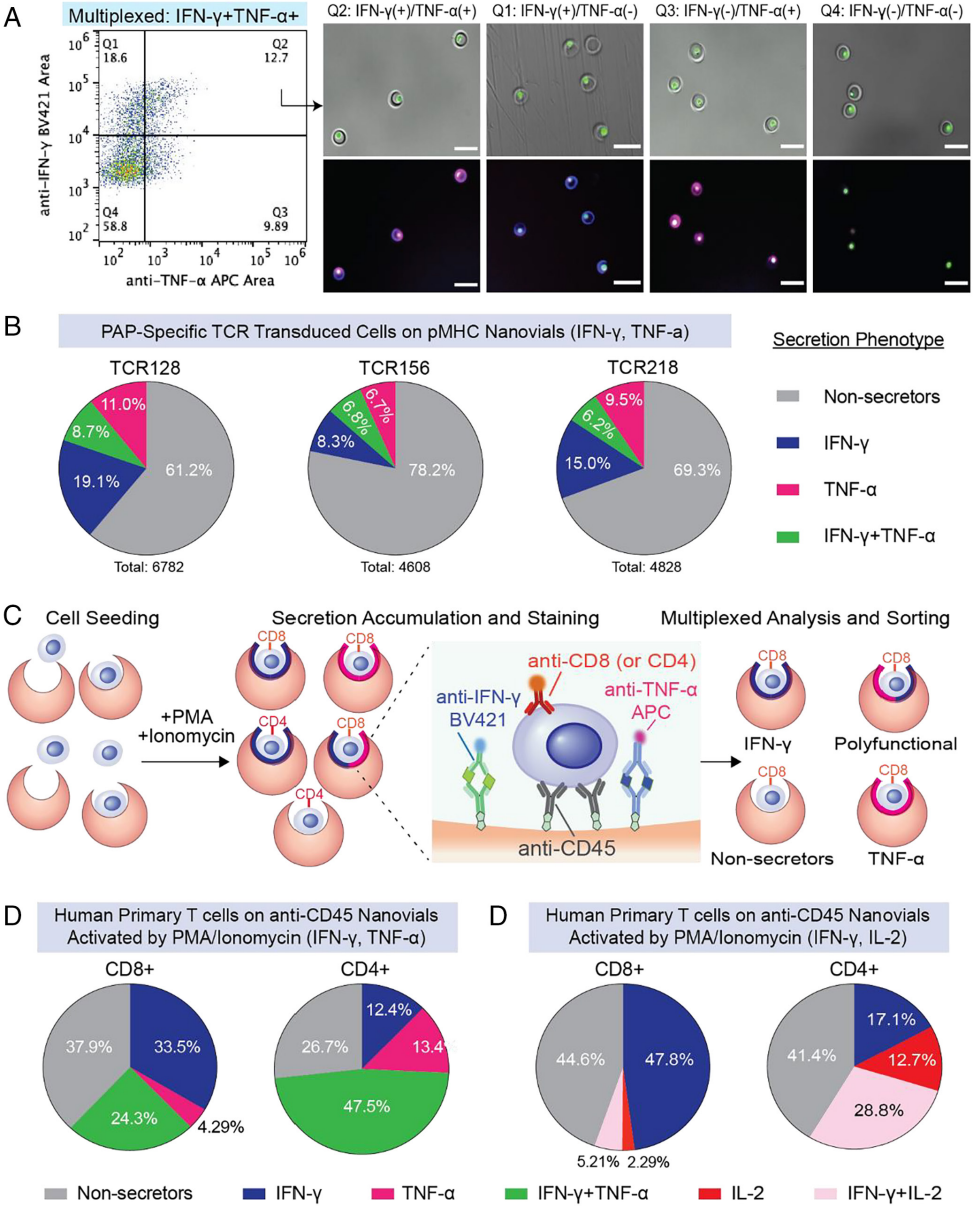
Multiplexed secretion-based profiling of prostate tissue antigen-specific T cells. (*A*) FACS analysis and sorting gates for identifying functional antigen-specific T cells transduced with TCR128 loaded on HLA-A*02:01 restricted PAP21 pMHC labeled nanovials. IFN-γ and TNF-α secretion signals were analyzed from the CD3+/CD8+/NGFR+ cells. Images of T cells on nanovials that were sorted reflecting each of the four quadrant gates including an IFN-γ and TNF-α polyfunctional population (Q2). (Scale bars represent 50 μm.) (*B*) The population distribution based on secretion phenotype is shown as a pie chart for TCR 128, 218, and 156 transduced cells. CD3+CD8+ cells with secretion signal below the background threshold were considered as nonsecretors. (*C*) Overview of multiplexed profiling of untransduced human primary T cells based on cytokine secretion and cell phenotype. T cells loaded on nanovials labeled with two cytokine capture antibodies (anti-IFN-γ and anti-TNF-α, or anti-IFN-γ and anti-IL-2) and anti-CD45 were activated under PMA/ionomycin stimulation. Secreted cytokines and cell surface markers (CD4, CD8) were stained with fluorescent detection antibodies, followed by analysis and sorting with a cell sorter. (*D*) The distribution of secretion phenotype for CD4+ and CD8+ human primary T cells based on IFN-γ and TNF-α secretion.

T cells engaged with APCs produce multiple cytokines simultaneously to achieve effector functions. As a proof of concept, we explored the capability of nanovials with additional anti-cytokine capture antibodies to profile multiple cytokines and link this secretion phenotype with surface markers. We first tested whether the multiplexed secretion assay can be applied to low-potency TCRs targeting PAP-specific antigens (19). In the context of HLA-A*02:01, TCR128 and 218 transduced PBMCs were loaded onto nanovials conjugated with PAP21 pMHC. TCR156 transduced PBMCs were loaded onto PAP22 pMHC labeled nanovials, and the noncognate PAP14 pMHC-nanovials acted as a negative control. Engineered CD3^+^CD8^+^ cells were highly enriched (NGFR^+^% > 90%) for all three tested PAP TCRs when loaded onto nanovials with their cognate pMHC (Fig. 5*A* and *SI Appendix*, Fig. S9 *A* and *B*) but not when using the nonspecific PAP14 pMHC or when loading untransduced cells (*SI Appendix*, Fig. S9 *C* and *D*). CD3^+^CD8^+^ cells representing a variety of secretion phenotypes, including an IFN-γ and TNF-α polyfunctional population, were successfully analyzed and sorted (Fig. 5*B*). The efficiency of nanovials in detecting multiple cytokines is similar for both strong tetramer signal (TCR128 and TCR218) and weak tetramer signal TCRs (TCR156) (19).

We further tested multiplexed-secretion profiling using untransduced human primary T cells activated with PMA and ionomycin coupled with CD8 and CD4 surface markers (Fig. 5*C*). We sorted populations of cells based on fluorescence peak areas exceeding the positive threshold for each individual cytokine as well as combinations of IFN-γ and TNF-α or IL-2 and IFN-γ (*SI Appendix*, Fig. S9 *E* and *F*). When measuring IFN-γ and TNF-α, the dominant secretion phenotype for CD8 cells was IFΝ-γ (33.5%) and only a small fraction of CD8+ cells secreted TNF-α alone (4.29%) (Fig. 5*D*). About 24% of CD8+ cells were polyfunctional, secreting both IFN-γ and TNF-α simultaneously. On the other hand, CD4+ cells had a larger polyfunctional population (47.5%) and this pattern was consistent when we analyzed for IFN-γ and IL-2 secretion (Fig. 5*D*). The multiplexed secretion profiling capability of nanovials could further improve the true identification rate of novel TCRs based on unique secretion phenotypes, as well as provide links to gene expression responsible for such polyfunctionality of each secreting cell.

## Discussion

Nanovials provide a tool to sort live antigen-specific T cells based on a combination of TCR binding and functional response (cytokine or granzyme B secretion) followed by recovery of reactive TCRs and epitope-specific annotation. This approach brings a number of advantages over conventional single-cell cognate T cell isolation platforms. First, nanovials can present pMHC at high density, providing an initial high avidity enrichment step from a large pool of cells (~20 million cells in our experiments). Even cells with low-affinity TCRs (5G6 and 9D2), which are not easily detectable using tetramer and dextramer staining, were recovered with higher purity. The ability to enrich a larger population of antigen-specific T cells than conventional duo-tetramers without a pre-expansion process not only reduces a week-long workflow into a single day but potentially enables rarer populations of cells to be identified. Nanovials were able to recover some previously reported CMV1- and EBV-specific TCR sequences (colored in *SI Appendix*, Fig. S10) along with a diverse set of unique TCR sequences that were validated to be functional (uncolored) (23, 24). This broader range does not come with the trade-off of low purity. High-purity screening is supported by the 78% functional hit rate of CMV and EBV-specific TCR clonotypes following re-expression where each target epitope was accurately identified. Other large-scaled pooled barcoded multimer approaches demonstrated a functional hit rate of ~50% or only assessed functionality of a few TCRs recovered instead of the entire set with 80% accuracy for calling matching epitopes (25, 26). Another study showed the ability to link TCR sequences with specific pMHC molecules using barcoded tetramers using single-cell sequencing in a multiwell plate format, although no data were presented on the fraction of the recovered TCR sequences that were reactive when re-expressed (27).

Using oligo-barcoded antibodies to label secreted cytokines allowed encoding of this cellular function into the single-cell sequencing dataset and ranking of TCR sequences based on the amount of cytokine(s) secreted. The ability to link TCR sequence information directly to secretion levels of cytokines also appears to improve the yield of functional TCRs following re-expression. We identified three functional TCRs that are prostate cancer specific, from a pool of 25 that were re-expressed. None of the highest frequency clonotypes (>3 of a clonotype) were functional upon re-expression. Notably, TCRs associated with the highest granzyme B secretion barcode signals (2 of 6, 33%) had the highest validation rate. As a comparison, in our previous study, we re-expressed 389 TCRs from more than 14 distinct healthy donors to retrieve 8 functional TCRs (2.05%) (12). Although the sample size is low, our results suggest secretion-based screening can dramatically improve the recovery rate for rare functional TCRs.

The accessibility and compatibility of nanovials with standard FACS and single-cell sequencing instrumentation can accelerate the development of personalized TCR immunotherapies. Epitopes for each recovered TCR are annotated through barcoding, while still being able to recover TCRs over a range of reactivity. Although we demonstrated only 10 different nanovial types, the number of pMHCs that can be multiplexed with nanovials is extensible to >40 based on commercial oligonucleotide-barcoding reagents, or ~1,000 using specialized manufacturing approaches (28). Since the TCR-pMHC interaction is heavily dependent on HLA-subtype restriction, the ability of nanovials to provide TCRs along with matching HLA-restricted epitopes leverages current technology limitations to simultaneously profile a large library of antigen-specific T cells, especially in disease models identified with diverse HLA genotypes like type 1 diabetes or COVID-19 (26, 29, 30).

By screening for TCRs based on the ability of cells to secrete a panel of cytokines, we can further explore links between TCR structure and cellular function and discover therapeutically important TCRs that, for example, are used by different cell subsets, such as regulatory T cells to prevent autoimmune conditions. The nanovial platform allows linking function, TCR sequence, and transcriptome at the single-cell level with high clarity, which can also further elucidate the role of TCRs across cytolytic and noncytolytic T cells. It is noteworthy that the MHC-nanovials employed in this study are, in theory, capable of providing only signal 1 in T cell activation (via pMHC-TCR interaction). Given the high-avidity effects provided by the ligand-coated nanovials, our current research is focused on incorporating costimulatory domains into the nanovials. This approach aims to develop an artificial antigen-presenting platform that can effectively stimulate naive T cell activation and proliferation. Recent work has emphasized the importance of functional characterization of TCRs, such as through assaying Ca^2+^ flux upon mechanical engagement of TCRs with pMHC-coated hydrogel beads, a platform that could be synergistic with nanovials to more fully functionally screen TCRs (31). We have shown that oligonucleotide-barcoded antibodies can be used as labels in the nanovial assay format [see also secretion-encoded single cell (SEC)-seq workflows] (32). These types of multiomic studies can ultimately uncover relationships between TCR structure and function for improved efficacy in T cell therapies. Beyond TCRs, the nanovial assay format should be applicable to other screening processes, e.g., for CAR-T cells, CAR-NK cells, TCR-mimics, or bispecific T cell engagers (BiTEs), with minor adjustments, opening up a new frontier in functional screening for cell therapy development.

## Materials and Methods

### Nanovial Fabrication.

Polyethylene glycol biotinylated nanovials with 35 μm diameters were fabricated using a three-inlet flow-focusing microfluidic droplet generator, sterilized and stored at 4 °C in Washing Buffer consisting of Dulbecco’s Phosphate Buffered Saline (Thermo Fisher) with 0.05% Pluronic F-127 (Sigma), 1% 1X antibiotic-antimycotic (Thermo Fisher), and 0.5% bovine serum albumin (Sigma) as previously reported (33).

### Nanovial Functionalization.

#### Streptavidin conjugation to the biotinylated cavity of nanovials.

Sterile nanovials were diluted in Washing Buffer five times the volume of the nanovials (i.e., 100 µL of nanovial volume was resuspended in 400 µL of Washing Buffer). A diluted nanovial suspension was incubated with equal volume of 200 μg/mL of streptavidin (Thermo Fisher) for 30 min at room temperature on a tube rotator. Excess streptavidin was washed out three times by pelleting nanovials at 2,000 × g for 30 s on a Galaxy MiniStar centrifuge (VWR), removing supernatant and adding 1 mL of fresh Washing Buffer.

#### Anti-CD45 and cytokine capture antibody labeled nanovials.

Streptavidin-coated nanovials were reconstituted at a five times dilution in Washing Buffer containing 140 nM (20 μg/mL) of each biotinylated antibody or cocktail of antibodies: anti-CD45 (Biolegend, 368534) and anti-IFN-γ (R&D Systems, BAF285), anti-TNF-α (R&D Systems, BAF210), anti-IL-2 (BD Sciences, 555040). Nanovials were incubated with antibodies for 30 min at room temperature on a rotator and washed three times as described above. Nanovials were resuspended at a five times dilution in Washing Buffer or culture medium prior to each experiment.

#### pMHC labeled nanovials.

MHC monomers with peptides of interest were synthesized and prepared according to a published protocol (34). Streptavidin-coated nanovials were reconstituted at a five times dilution in Washing Buffer containing 20 μg/mL biotinylated pMHC and 140 nM of anti-IFN-γ antibody or 140 nM of anti-granzyme B antibody (R&D systems, BAF2906) unless stated otherwise. For oligonucleotide barcoded nanovials, 1 µL of 0.5 mg/mL totalseq-C streptavidin (Biolegend, 405271, 405273, 405275) per 6 µL nanovial volume was additionally added during the streptavidin conjugation step.

### Cell Culture.

#### Human primary T cells.

Human primary T cells were cultured as previously reported (33).

#### Human donor PBMCs.

To prime naive T cells with peptides of interest, PBMCs from commercial vendors (AllCells) were cultured and processed as previously described with chemically synthesized peptides (>80% purity, Elim Biopharm) (19).

#### K562 and Jurkat-NFAT-ZsGreen.

K562 (ATCC) and Jurkat-NFAT-ZsGreen (gift from D. Baltimore at Caltech) were cultured in RPMI 1640 (Thermo Fisher) with 10% FBS (Omega Scientific) and Glutamine (Fisher Scientific). 293T (ATCC) was cultured in DMEM (Thermo Fisher) with 10% FBS and Glutamine.

### Isolation of Viral Epitope-specific T Cells using Nanovials, Tetramers, and CD137 Staining.

Nanovials were functionalized with HLA-A*02:01 restricted pMHCs targeting cytomegalovirus pp65, cytomegalovirus IE1 or Eptsein-Barr virus BMLF1 with corresponding totalseq-C streptavidin barcodes C0971, C0972, C0973 (Biolegend, 405271, 405273, 405275) as described above. All sets of functionalized nanovials were pooled together as one nanovial suspension (a total of 0.75 million nanovials). PBMCs were activated for 7 d with peptides associated with each antigen (CMV1: pp65/NLVPMVATV, CMV2: IE1/VLEETSVML, EBV: BMLF1/GLCTLVAML) as reported previously (19). Five million activated PBMCs were loaded onto the pooled nanovial suspension. Following recovery and activation on nanovials for 3 h, samples were stained with viability dye and a cocktail of detection antibodies (calcein AM, anti-CD3 APC Cy7, anti-CD8 PE, anti-IFN-γ). Using a cell sorter, viable CD3 and CD8 cells on nanovials with IFN-γ secretion signal were sorted. In parallel, five million activated PBMCs were each stained with a surface activation marker (CD137) or CMV1 pMHC tetramers and sorted as previously reported (9). All sorted samples were reconstituted in 18 µL of 1× PBS containing 0.04% BSA.

### Direct Enrichment of Antigen-specific T Cells without Preactivation Process.

Nanovials were functionalized with HLA-A*02:01 restricted CMV pp65 (NLVPMVATV) pMHCs and anti-IFN-γ antibody. Then, 10^7^ freshly thawed PBMCs were directly loaded onto nanovials without 7 d of preactivation with CMV pp65 peptide. Following recovery and activation on nanovials for 3 h, samples were stained with detection antibody cocktail containing calcein AM, anti-CD3 APC Cy7, anti-CD8 PE, and anti-IFN-γ at concentration described in *SI Appendix*, Table S1. In parallel, 10^7^ of the same PBMCs were stained with anti-CD3 PerCp Cy5.5, anti-CD8 PE, and CMV pp65 tetramer as previously reported (9). Samples were analyzed using a cell sorter by gating to CD3+CD8+ cells on nanovials with IFN-γ signal or to CD3+CD8+ cells with tetramer signal.

### Nanovial-based Isolation of Prostate Cancer Epitope-specific T Cells from One Donor.

Nanovials were functionalized with anti-granzyme B antibody and HLA-A*02:01 restricted pMHCs each targeting 10 different prostate acid phosphatase (PAP) and cancer-enhanced splicing epitopes discovered in previous study (19). Oligonucleotide streptavidin barcode was also added to encode each pMHC molecule on nanovials. PBMCs from one healthy donor were preactivated for 7 days with peptides associated with each antigen: PAP14 (ILLWQPIPV), PAP21 (LLLARAASLSL), PAP22 (TLMSAMTNL), PAP23 (LLFFWLDRSVLA), CTNND1 (MQDEGQESL), CLASP1 (SLDGTTTKA), MEAF6 (SGMFDYDFEYV), PXDN (HLFDSVFRFL), SCAMP3 (STMYYLWML), and TCF12 (SLHSLKNRV). Twenty million activated PBMCs were loaded onto the pooled nanovial suspension. Following recovery and activation on nanovials for 3 h, samples were stained with viability dye and a cocktail of detection antibodies (calcein AM, anti-CD3 APC Cy7, anti-CD8 PE, anti-granzyme B APC). After washing, samples were also incubated with oligonucleotide anti-APC antibody. Using a cell sorter, viable CD3+CD8+ cells on nanovials with granzyme B signal were sorted (35).

### Functional Validation of Recovered TCR Sequences.

To measure antigen-specific reactivity of recovered CMV-,EBV-, or cancer epitope-specific TCR sequences, TCRs were expressed and screened in Jurkat-NFAT-GFP cells as described previously (36). Jurkat-NFAT-GFP cells containing multiple NFAT-binding motifs followed by GFP genes were used as the reporter cell line. Paired TCRs with murine constant regions were introduced into Jurkat-NFAT-GFP cells via electroporation. Upon TCR-MHC recognition, NFAT will drive the transcription of GFP and can then be quantified and analyzed by FACS. Murine TCR constant regions were also analyzed by FACS to ensure TCR surface presentation.

Paired TCR alpha and beta chains of interest were cloned into a retroviral pMSGV construct as previously described (17). PBMCs for retroviral transduction were processed and cultured according to our recent publication (19, 36). Briefly, healthy donor PBMCs were activated by CD3/CD28 dynabeads followed by retroviral infection with TCRs of interest. Truncated NGFR was used as a cotransduction marker. Constant regions of TCR alpha and beta chains were replaced with mouse counterparts to minimize mispairing in human T cells. Total T cell population (both CD8^+^ and CD4^+^) was analyzed 7 days postinfection by FACS to ensure high-quality infection (50 to 70% NGFR^+^). Similar to the Jurkat-NFAT-GFP assay, murine TCR constant regions were also tested to ensure correct membrane allocation in human PBMCs. No significant transduction and membrane allocation differences were found among the candidate TCRs tested in this study.

To assess the function of the transduced TCRs in human PBMCs, TCR-expressing cells were mixed with K562-A2 cells at a ratio of 1:2 (Effector:Target) in the RPMI media and supplemented with 1 µg/mL of anti-CD28/CD49d antibodies (BD Biosciences, 347690) and 1 µg/mL of cognate peptides or mixed peptide library. For PBMCs, supernatants were collected after 48 h and analyzed by ELISA (BD Biosciences) to estimate IFN-γ concentration. PBMCs transduced by the vector without a TCR was used as a negative control.

### Multiplexed Secretion–based Profiling to Identify Polyfunctional T Cells.

#### Linking cell surface markers to secretion phenotype.

Streptavidin-coated nanovials were decorated with biotinylated antibodies (140 nM of anti-CD45, anti-IFN-γ and anti-TNF-α or anti-CD45, anti-IFN-γ and 140 nM anti-IL-2). Negative control nanovials were prepared by labeling nanovials only with anti-CD45 antibody without any cytokine capture antibodies. Then, 0.5 million human primary T cells were loaded onto nanovials and recovered in T cell expansion medium containing 10 ng/mL PMA and 500 ng/mL ionomycin. Following 3 h of activation, secreted cytokines were stained with fluorescent detection antibodies (anti-IFN-γ BV421, anti-TNF-α APC, and anti-IL-2 APC) and cells were stained with 0.3 μM calcein AM, 5 µL of 25 μg/mL anti-CD4 PE (Biolegend, 344606) and 5 µL of 100 μg/mL anti-CD8 Alexa Fluor 488 (Biolegend, 344716) per 6 µL nanovial volume. Using a cell sorter, CD4 or CD8 cells on nanovials with secretion signal were evaluated by first creating quadrant gates based on the negative control sample (nanovials only labeled with anti-CD45 antibody). Q1 was defined as nanovials with only IFN-γ secreting cells. Q2 was nanovials with polyfunctional T cells that secreted both cytokines (IFN-γ and TNF-α or IL-2). Q3 was nanovials with either TNF-α or IL-2 secreting cells while Q4 was nanovials with nonsecretors. Nanovials in each quadrant were sorted and imaged with a fluorescence microscope to quantify enrichment of each cell type and their associated secretion characteristics.

#### Multiplexed secretion-based profiling of cancer-specific cognate T cells.

PBMCs were transduced with prostate acid phosphatase-specific TCRs (TCR128, 156, 218) as previously described (19). Streptavidin-coated nanovials were functionalized with biotinylated anti-IFN-γ, anti-TNF-α, and pMHC targeting each TCR: PAP21 for both TCR128 and TCR218, and PAP22 for TCR156. As a negative control, noncognate pMHC (PAP14 for TCR156) labeled nanovials were also prepared. PBMCs transduced with each TCR were separately loaded onto nanovials and activated for 3 h, followed by secondary staining with anti-CD3 PerCp Cy5.5, anti-CD8 PE, anti-IFN-γ ΒV421, anti-TNF-α APC, anti-NGFR PE Cy7, and calcein AM. Using a cell sorter, CD3, CD8, and NGFR positive cells on nanovials with each secretion phenotype (IFN-γ, TNF-α, polyfunctional, nonsecretors) were sorted and imaged using a fluorescence microscope.

## Supplementary Material

Appendix 01 (PDF)

## Data Availability

The data discussed in this publication have been deposited in NCBI’s Gene Expression Omnibus (37) and are accessible through GEO Series accession number GSE252830 (https://www.ncbi.nlm.nih.gov/geo/query/acc.cgi?acc=GSE252830). All other data are included in the manuscript and/or *SI Appendix*.
