# Supplementary material for: Defining T cell receptor repertoires using nanovial-based binding and functional screening
Source: Proc Natl Acad Sci U S A. 2024 Mar 27;121(14):e2320442121. doi: 10.1073/pnas.2320442121 (PMC10998554; doi:10.1073/pnas.2320442121)
Supplement: Supplementary file 1 — Appendix 01 (PDF) [file pnas.2320442121.sapp.pdf]

Supplementary Materials:

Supplementary Figure 1. Nanovial fabrication and functionalization.

Supplementary Figure 2. Optimization of human primary T cell loading.

Supplementary Figure 3. Selection of viable T cells based on cytokine secretion.

Supplementary Figure 4. Optimization of antigen-specific T cell loading, secretion on nanovials and expansion of cells post-isolation.

Supplementary Figure 5. Recovery of cognate T cells with various affinities to HLA-A*02:01 restricted NY-ESO-1 pMHC.

Supplementary Figure 6. FACS analysis and gating strategy for isolation of cognate T cells using nanovials, tetramers or CD137 activation markers.

Supplementary Figure 7. Determination of the dominant epitope for each clonotype from the number of barcodes detected.

Supplementary Figure 8. Linking cell secretion to recovered TCRs and their functional validation.

Supplementary Figure 9. Detailed FACS analysis and gating strategy for multiplexed secretion-based profiling of antigen-specific T cells.

Supplementary Figure 10. Detailed list of clonotypes recovered from nanovials (frequency >=2) with corresponding V(D)J αβ genes, CDR3 αβ sequences.

Supplementary Figure 11. Heatmap of GFP positivity from each recovered TCR upon re-expression in a GFP reporter line and exposure to antigen presenting cells with and without cognate peptides.

Supplementary Note Figure: Area vs. Height Gating for Secretion Detection.

Supplementary Materials and Methods

Supplementary References

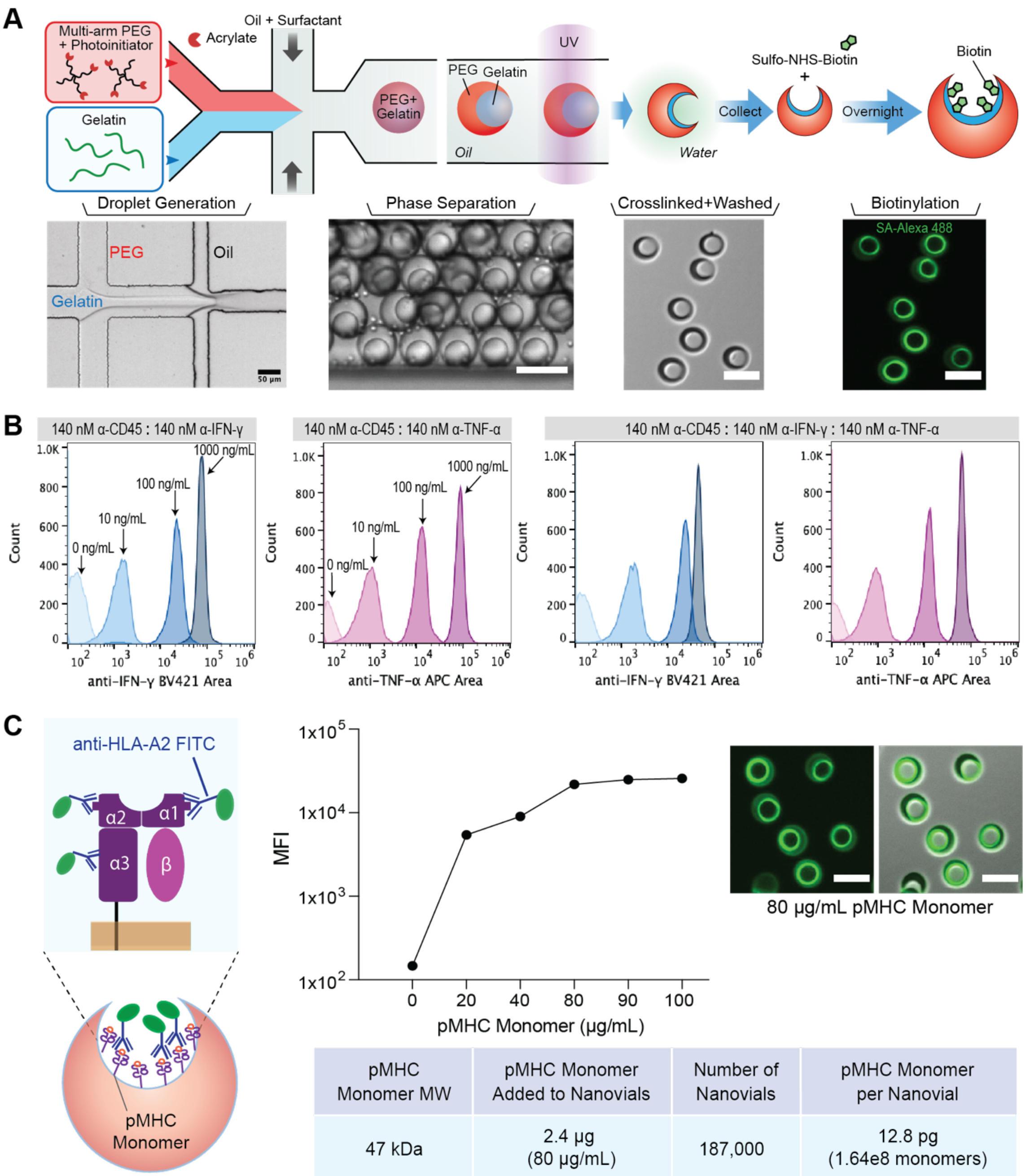

pMHC Monomer MW	pMHC Monomer Added to Nanovials	Number of Nanovials	pMHC Monomer per Nanovial
47 kDa	2.4 μg (80 μg/mL)	187,000	12.8 pg (1.64e8 monomers)

Supplementary Figure 1: Nanovial fabrication and functionalization. (A) An aqueous phase comprised of 4-arm-PEG Acrylate and photoinitiator is co-flowed with a gelatin solution in a microfluidic droplet generator. After droplet formation, PEG and gelatin undergo phase separation and are exposed to UV light to cross-link. After collection, nanovials are incubated with sulfo-NHS-biotin to be biotinylated. Localized fluorescence of AlexaFluor488-labeled streptavidin is observed in nanovial cavities. Scale bar represents 50 μm. (B) Flow cytometry fluorescence histograms of nanovials following a dose-dependent cytokine capture assay with different capture antibody combinations. Nanovials were functionalized with anti-CD45 and with one or two cytokine capture antibodies (anti-IFN-γ, anti-TNF-α) and incubated with 0, 10, 100, or 1000 ng/mL of recombinant TNF-α or IFN-γ. The ability of nanovials to detect each individual cytokine was not significantly affected by the presence of other cytokine capture antibodies. (C) Fluorescence intensity

based on the concentration of pMHC incubated with nanovials. Nanovials were incubated with 0-100 μg/mL of pMHC monomers and stained with fluorescent anti-HLA-A2 antibodies followed by measurement of fluorescence intensity via SONY SH800 sorter. Signal of loaded pMHC increases linearly up to a concentration of ~80 μg/mL of pMHC monomers. Scale bars represent 50 μm.

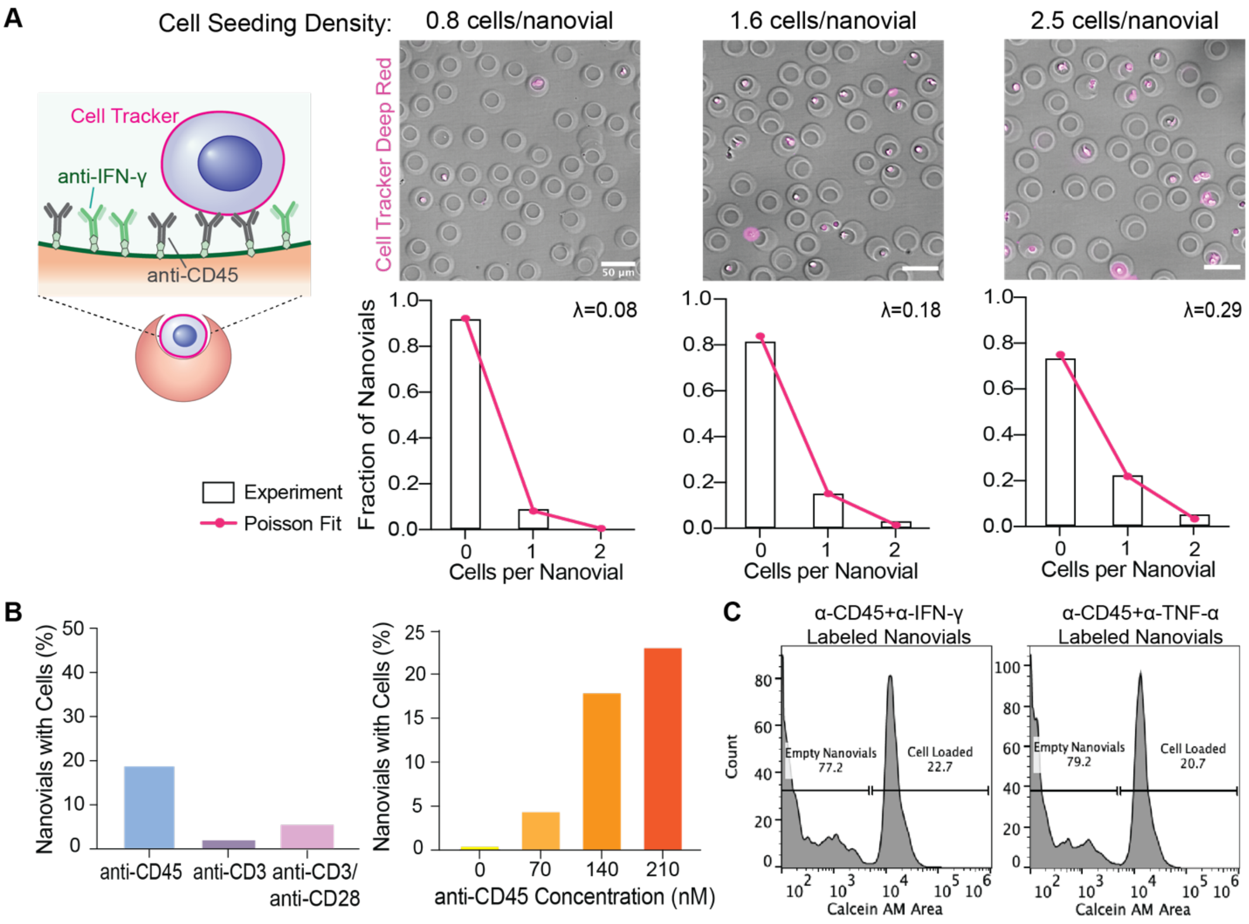

Supplementary Figure 2: Optimization of human primary T cell loading. (A) Loading of human primary T cells into anti-CD45 and anti-IFN-γ labeled nanovials at different cell number to nanovial ratios. Using custom image analysis algorithm in MATLAB, the total number of nanovials with 0, 1, 2 or more cells in each image frame were measured (n>2000). Replicates of each sample were analyzed. The highest fraction of single-cell loaded nanovials was achieved when cells were seeded at 1.6 cells per nanovial. Increased cell seeding density resulted in a larger fraction of nanovials with two or more T cells. Loading of cells into nanovial cavities followed Poisson statistics. Scale bars represent 50 µm. (B) Dependence of cell binding on surface marker target and antibody concentration. Left: Nanovials conjugated with anti-CD45 showed the highest loading efficiency when primary T cells were loaded after initial CD3/CD28 activation in culture. Right: Increased anti-CD45 concentration on nanovials increased cell binding by nearly 6-fold. (C) Effect of cytokine capture antibodies on cell loading. Flow cytometry histograms of cells (calcein AM positive) loaded on nanovials in the presence of anti-IFN-γ or anti-TNF-α antibodies.

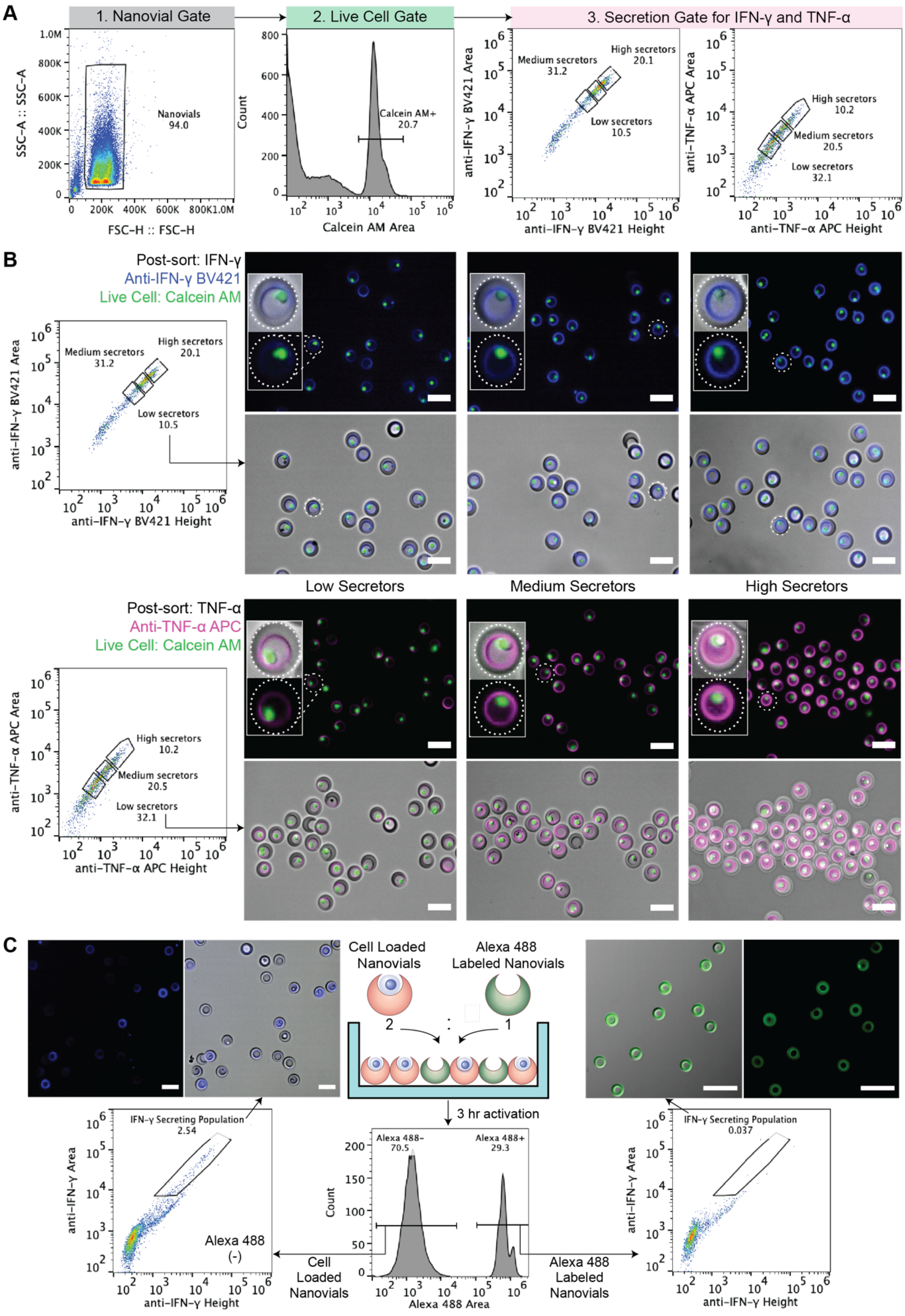
A
1. Nanovial Gate
2. Live Cell Gate
3. Secretion Gate for IFN-γ and TNF-α
SSC-A :: SSC-A
FSC-H :: FSC-H
Nanovials 94.0
Count
Calcein AM Area
Calcein AM+ 20.7
anti-IFN-γ BV421 Area
anti-IFN-γ BV421 Height
Medium secretors 31.2
High secretors 20.1
Low secretors 10.5
anti-TNF-α APC Area
anti-TNF-α APC Height
High secretors 10.2
Medium secretors 20.5
Low secretors 32.1
B
Post-sort: IFN-γ
Anti-IFN-γ BV421
Live Cell: Calcein AM
Low Secretors
Medium Secretors
High Secretors
Post-sort: TNF-α
Anti-TNF-α APC
Live Cell: Calcein AM
C
Cell Loaded Nanovials
Alexa 488 Labeled Nanovials
2
1
3 hr activation
IFN-γ Secreting Population 2.54
anti-IFN-γ Area
anti-IFN-γ Height
Alexa 488 (-)
Alexa 488- 70.5
Alexa 488+ 29.3
Alexa 488 Area
Cell Loaded Nanovials
Alexa 488 Labeled Nanovials
IFN-γ Secreting Population 0.037

Supplementary Figure 3: Selection of viable T cells based on cytokine secretion. (A) Gates for isolating cells on nanovials with secretion signal. Using a SONY SH800S, the nanovial population was identified in FSC/SSC and further gated for high calcein AM signal. Secretion signal was quantified for this sub-population using the fluorescence peak area vs. peak height (A/H). Single cells were sorted as high, medium, and low secretors based on IFN-γ and TNF-α secretion level. (B) Fluorescence microscopy images of sorted high, medium, and low secretors. Dotted lines in insets outline the nanovial boundaries. Scale bars represent 50 μm. (C) Crosstalk between nanovials. Two nanovial types were introduced together to evaluate crosstalk. Fluorescently labeled nanovials (AlexaFluor 488) without cells were mixed with T cell-loaded nanovials activated with PMA/ionomycin at a ratio of 1:2. Secretion signal was labeled and analyzed on both nanovial types by first gating on the green fluorescence signal on nanovials (Alexa 488 Area). Scatter plots of the cell-loaded nanovial population shows a larger population with high IFN-γ secreting cells compared to nanovials that were not loaded with cells. The percent of cells in the identical gated regions are shown. Scale bars represent 100 μm.

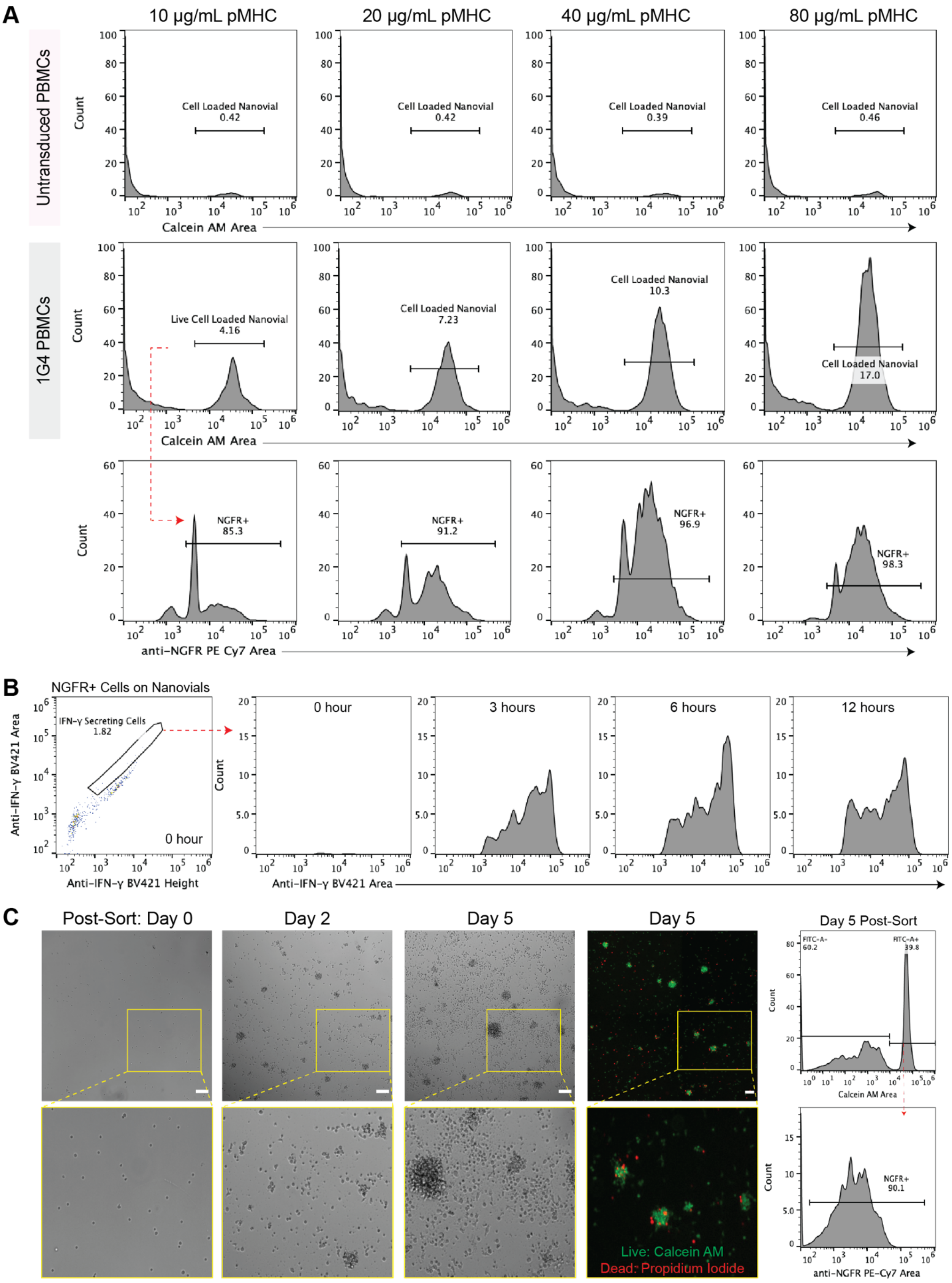
A
10 µg/mL pMHC
20 µg/mL pMHC
40 µg/mL pMHC
80 µg/mL pMHC
Untransduced PBMCs
Cell Loaded Nanovial 0.42
Cell Loaded Nanovial 0.42
Cell Loaded Nanovial 0.39
Cell Loaded Nanovial 0.46
Calcein AM Area
1G4 PBMCs
Live Cell Loaded Nanovial 4.16
Cell Loaded Nanovial 7.23
Cell Loaded Nanovial 10.3
Cell Loaded Nanovial 17.0
Calcein AM Area
NGFR+ 85.3
NGFR+ 91.2
NGFR+ 96.9
NGFR+ 98.3
anti-NGFR PE Cy7 Area
Count
B
NGFR+ Cells on Nanovials
IFN-γ Secreting Cells 1.82
0 hour
Anti-IFN-γ BV421 Area
Anti-IFN-γ BV421 Height
0 hour
3 hours
6 hours
12 hours
Anti-IFN-γ BV421 Area
C
Post-Sort: Day 0
Day 2
Day 5
Day 5
Live: Calcein AM
Dead: Propidium Iodide
Day 5 Post-Sort
FITC-A- 60.2
FITC-A+ 39.8
Calcein AM Area
NGFR+ 90.1
anti-NGFR PE-Cy7 Area

Supplementary Figure 4: Optimization of antigen-specific T cell loading, secretion on nanovials and expansion of cells post-isolation. PBMCs were activated by dynabeads to induce expansion. Dynabeads were removed at least 48 hour prior to loading onto nanovials to minimize non-specific cytokine release. (A) Analysis of antigen-specific T cell loading as a function of pMHC concentrations on nanovials. Top flow cytometry histograms show the fraction of untransduced cell loaded nanovials based on calcein AM signal. Bottom shows the fraction of 1G4-transduced cell nanovials and corresponding NGFR positivity of those bound cells (red dashed line). (B) Flow cytometry fluorescence histograms of nanovials following loading of 1G4-transduced PBMCs on pMHC-labeled nanovials and secretion accumulation at different time points. The leftmost panel of the flow cytometry plot represents calcein AM+NGFR+ cell loaded onto nanovials and stained for IFN-γ signal at 0 hour time point. The first histogram shows IFN-γ signal at 0 hour time point from "IFN-γ secreting cell gate". At 3, 6, 12 hour time points, IFN-γ signal was also analyzed from calcein AM+NGFR+ cell-loaded nanovials and represented as histograms, showing increase in IFN-γ signal over time. (C) Images showing expansion of 1G4-transduced T cells following sorting and detachment with collagenase D treatment. Microscopy images show cells expanded in culture over 5 days. (Right) Flow cytometry fluorescence histogram of viability and NGFR levels for the expanded population expressing the 1G4 TCR on Day 5. Scale bars represent 50 µm.

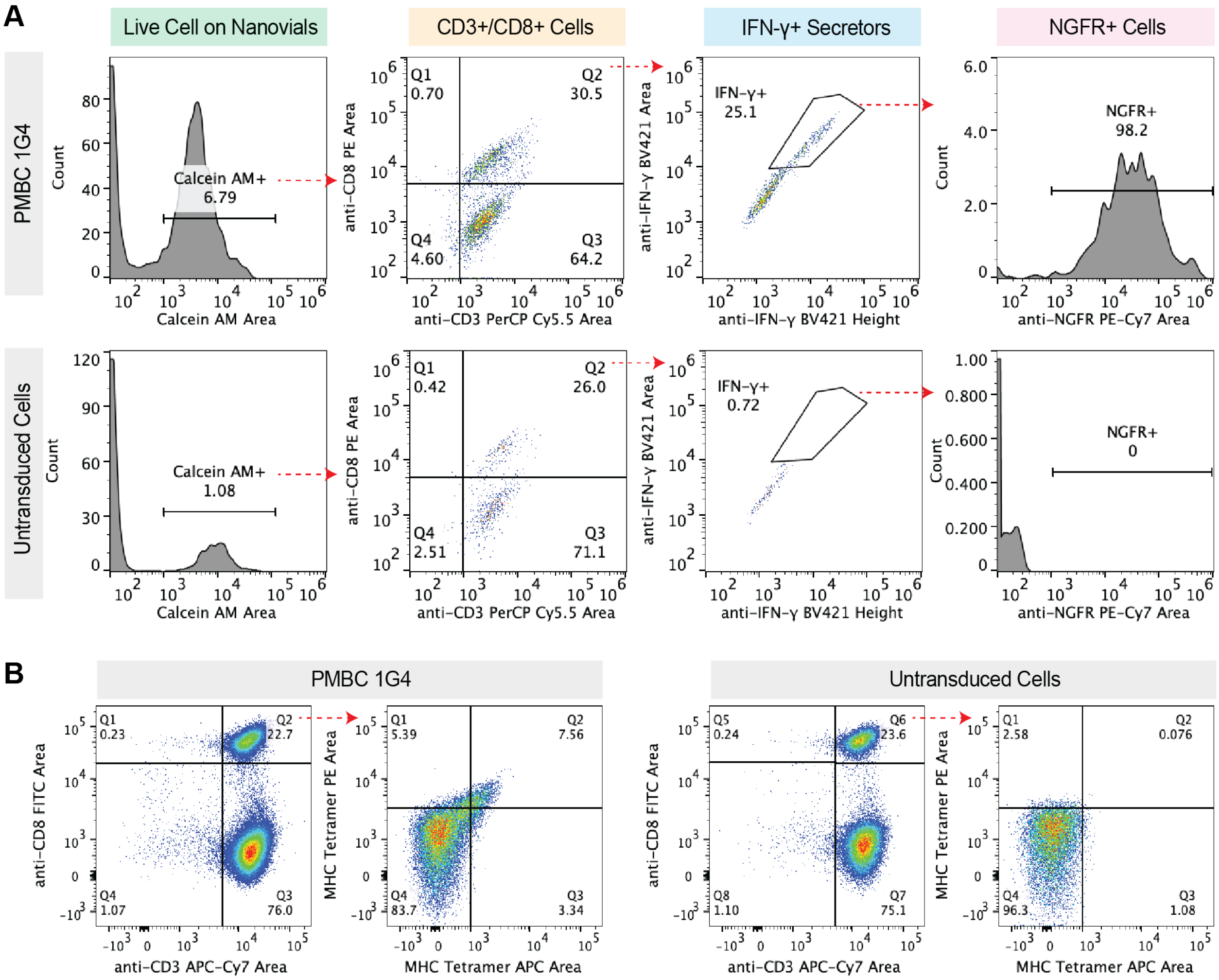

C

TCR Transduced PBMCs	Tetramers	Nanovials	
	CD3+CD8+Tetramer+NGFR+	CD3+CD8+NGFR+	CD3+CD8+IFN-γ+NGFR+
3A1	4017	1874	653
1G4	1797	1775	571
4A2	15	1996	447
5G6	98	2222	888
9D2	9	953	362
UT	0	0	0

Supplementary Figure 5: Recovery of cognate T cells with various affinities to HLA-A*02:01 restricted NY-ESO-1 pMHC are shown. (A) Flow cytometry plots of isolating 1G4-transduced PBMCs or untransduced cells loaded onto NY-ESO-1 pMHC labeled nanovials. Enriched cells were defined based on gates on viability (calcein AM), CD3 and CD8, and IFN-γ secretion. The NGFR fraction above the background was also determined using a gate on PE-Cy7 area. (B) Flow cytometry plots of isolating 1G4-transduced PBMCs

or untransduced cells based on dual-color MHC tetramer signal. (C) Raw numbers of recovered cognate T cells with various affinities using pMHC-labeled nanovials or dual-color tetramers (based on one experiment).

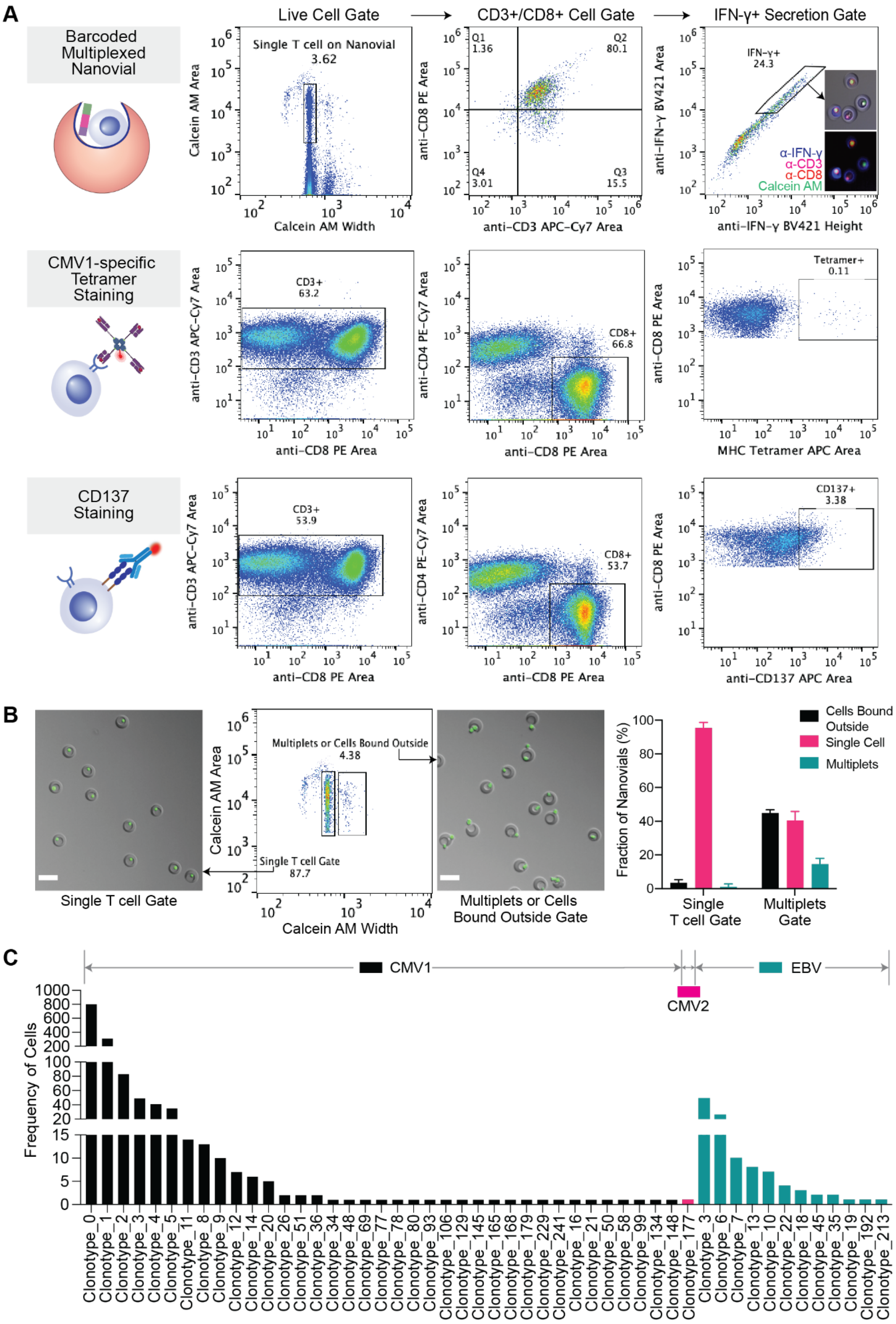
A
Barcoded Multiplexed Nanovial
Live Cell Gate
CD3+/CD8+ Cell Gate
IFN-γ+ Secretion Gate
Single T cell on Nanovial 3.62
Calcein AM Area
Calcein AM Width
anti-CD8 PE Area
anti-CD3 APC-Cy7 Area
Q1 1.36
Q2 80.1
Q3 15.5
Q4 3.01
anti-IFN-γ BV421 Area
anti-IFN-γ BV421 Height
IFN-γ+ 24.3
α-IFN-γ
α-CD3
α-CD8
Calcein AM
CMV1-specific Tetramer Staining
CD3+ 63.2
anti-CD3 APC-Cy7 Area
anti-CD8 PE Area
CD8+ 66.8
anti-CD4 PE-Cy7 Area
Tetramer+ 0.11
MHC Tetramer APC Area
CD137 Staining
CD3+ 53.9
CD8+ 53.7
CD137+ 3.38
anti-CD137 APC Area
B
Single T cell Gate
Multiplets or Cells Bound Outside 4.38
Single T cell Gate 87.7
Calcein AM Width
Multiplets or Cells Bound Outside Gate
Fraction of Nanovials (%)
Cells Bound Outside
Single Cell
Multiplets
Single T cell Gate
Multiplets Gate
C
CMV1
CMV2
EBV
Frequency of Cells
Clonotype_0
Clonotype_1
Clonotype_2
Clonotype_3
Clonotype_4
Clonotype_5
Clonotype_11
Clonotype_8
Clonotype_9
Clonotype_12
Clonotype_14
Clonotype_20
Clonotype_26
Clonotype_51
Clonotype_36
Clonotype_34
Clonotype_48
Clonotype_69
Clonotype_77
Clonotype_78
Clonotype_80
Clonotype_93
Clonotype_106
Clonotype_129
Clonotype_145
Clonotype_165
Clonotype_168
Clonotype_179
Clonotype_229
Clonotype_241
Clonotype_16
Clonotype_21
Clonotype_50
Clonotype_58
Clonotype_99
Clonotype_134
Clonotype_148
Clonotype_177
Clonotype_3
Clonotype_6
Clonotype_7
Clonotype_13
Clonotype_10
Clonotype_22
Clonotype_18
Clonotype_45
Clonotype_35
Clonotype_19
Clonotype_192
Clonotype_213

Supplementary Figure 6: FACS analysis and gating strategy for isolation of cognate T cells using nanovials, tetramers, or CD137 activation markers. (A) Flow scatter plots showing gates for selecting viable CD8+ T cells binding to nanovials and secreting IFN-γ, CD137+ CD8+ T cells, and tetramer+ CD8+ T cells. Microscopy images show representative sorted cells on nanovials. Scale bar represents 50 μm. (B) Scatter plots show calcein AM fluorescence Peak Area vs. Peak Width gates used for isolating single cells on nanovials. Images of sorted events show that the population with a similar peak area but with a shorter peak width contains >90% single cells loaded on nanovials. Data on single-cell occupancy is shown in a bar graph. Scale bars represent 50 μm. (C) Distribution of cell clonotypes recovered by nanovials color coded with matching target pMHC information: 32 CMV1 (black), 1 CMV2 (magenta), 12 EBV (green) specific clonotypes.

Clonotype_id	Frequency	Number of Barcodes Detected	Dominant Epitope	Dominant Epitope (%)
clonotype0	801	CMV1(597), EBV(10), CMV2(12)	CMV1	96.4
clonotype1	308	CMV1(187), EBV(2), CMV2(7)	CMV1	95.4
clonotype2	83	CMV1(29), CMV2(2), EBV(1)	CMV1	90.6
clonotype3	49	EBV(36), CMV1(3)	EBV	92.3
clonotype4	41	CMV1(31), EBV(1), CMV2(1)	CMV1	93.9
clonotype5	35	CMV1(26)	CMV1	100.0
clonotype6	26	EBV(21), CMV1(2)	EBV	91.3
clonotype11	14	CMV1(13)	CMV1	100.0
clonotype8	13	CMV1(3)	CMV1	100.0
clonotype9	10	CMV1(6)	CMV1	100.0
clonotype7	10	EBV(10)	EBV	100.0
clonotype13	8	EBV(5)	EBV	100.0
clonotype12	7	CMV1(5)	CMV1	100.0
clonotype10	7	EBV(6)	EBV	100.0
clonotype14	6	CMV1(5)	CMV1	100.0
clonotype20	5	CMV1(3)	CMV1	100.0
clonotype22	4	EBV(2)	EBV	100.0
clonotype18	3	EBV(2)	EBV	100.0
clonotype26	2	CMV1(1)	CMV1	100.0
clonotype51	2	CMV1(2)	CMV1	100.0
clonotype45	2	EBV(1)	EBV	100.0
clonotype35	2	EBV(2)	EBV	100.0
clonotype36	2	CMV1(1)	CMV1	100.0
clonotype34	1	CMV1(1)	CMV1	100.0
clonotype48	1	CMV1(1)	CMV1	100.0
clonotype69	1	CMV1(1)	CMV1	100.0
clonotype77	1	CMV1(1)	CMV1	100.0
clonotype78	1	CMV1(1)	CMV1	100.0
clonotype80	1	CMV1(1)	CMV1	100.0
clonotype93	1	CMV1(1)	CMV1	100.0
clonotype106	1	CMV1(1)	CMV1	100.0
clonotype129	1	CMV1(1)	CMV1	100.0
clonotype145	1	CMV1(1)	CMV1	100.0
clonotype165	1	CMV1(1)	CMV1	100.0
clonotype168	1	CMV1(1)	CMV1	100.0
clonotype179	1	CMV1(1)	CMV1	100.0
clonotype229	1	CMV1(1)	CMV1	100.0
clonotype241	1	CMV1(1)	CMV1	100.0
clonotype16	1	CMV1(2)	CMV1	100.0
clonotype19	1	EBV(1)	EBV	100.0
clonotype21	1	EBV(1)	EBV	100.0
clonotype50	1	EBV(1)	EBV	100.0
clonotype58	1	EBV(1)	EBV	100.0
clonotype99	1	EBV(1)	EBV	100.0
clonotype134	1	EBV(1)	EBV	100.0
clonotype148	1	EBV(1)	EBV	100.0
clonotype177	1	CMV2(1)	CMV2	100.0
clonotype192	1	EBV(1)	EBV	100.0
clonotype213	1	EBV(1)	EBV	100.0

Supplementary Figure 7: Determination of the dominant epitope for each clonotype from the number of barcodes detected. For clonotypes matched with more than one barcode (clonotype0, 1, 2, 3, 4, 6), the final epitope was determined based on the barcode with the highest fraction, representing >90% of cells represented.

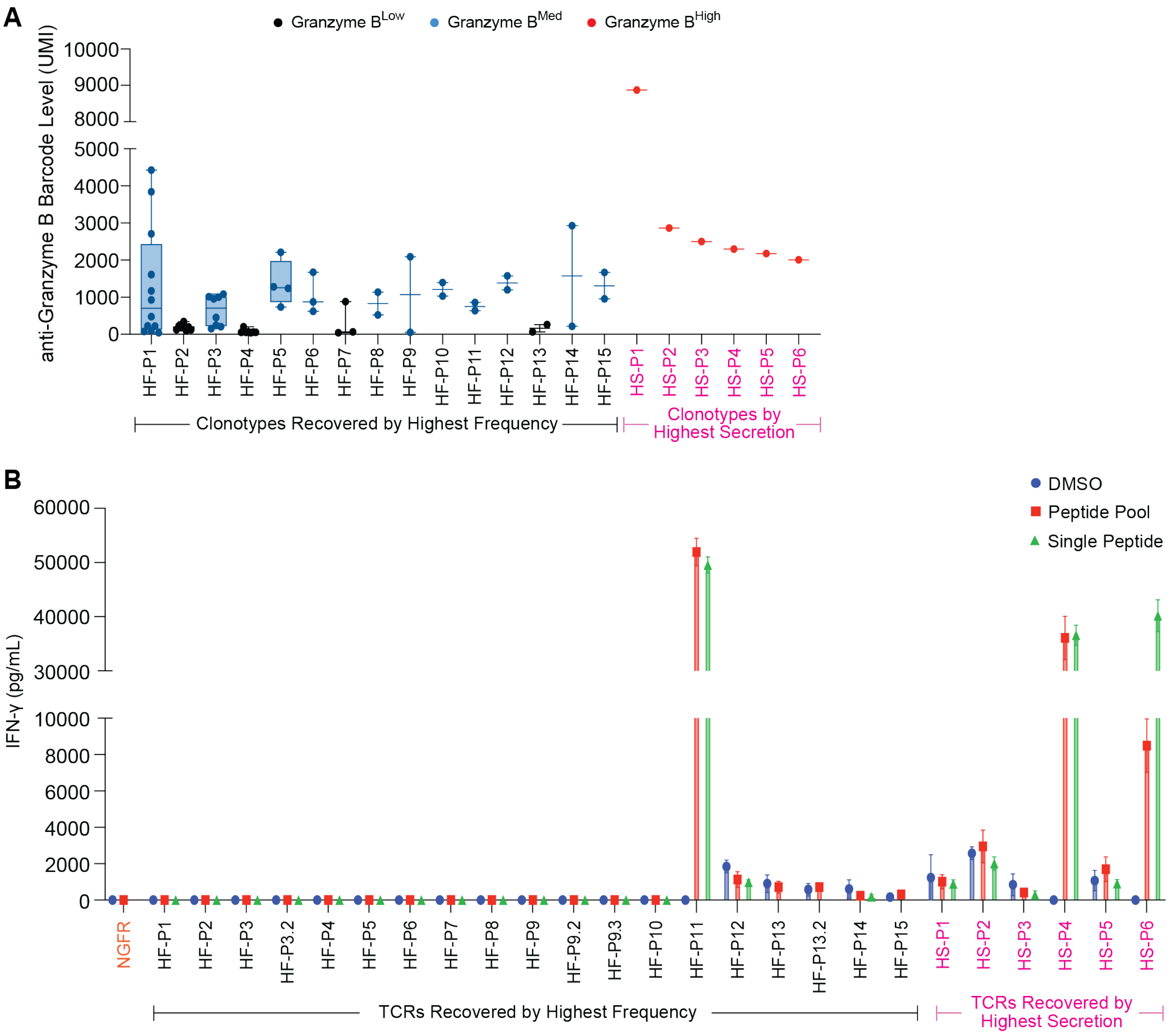

Supplementary Figure 8: Linking cell secretion to recovered TCRs and their functional validation. (A) Clonotypes were ranked based on granzyme B secretion barcode signal (pink, HS-P1 to HS-P6) or frequency of recovered TCR sequence (black, HF-P1 to HF-P15). Each clonotype was classified as Granzyme B^{High} (Red dots), Granzyme B^{Medium} (Blue dots) or Granzyme B^{Low} (Black dots) based on their secretion level (Figure 4D). (B) Re-expression of each candidate TCR sequence in human PBMCs and measurement of IFN-γ secretion following exposure to antigen presenting cells with exogenously added cognate peptides: peptide pool (red bars) or single-peptide obtained from nanovial barcode encoding the specific pMHC molecule (green bars). As a negative control, each TCR-transduced PBMC pool was co-cultured with antigen presenting cells with DMSO.

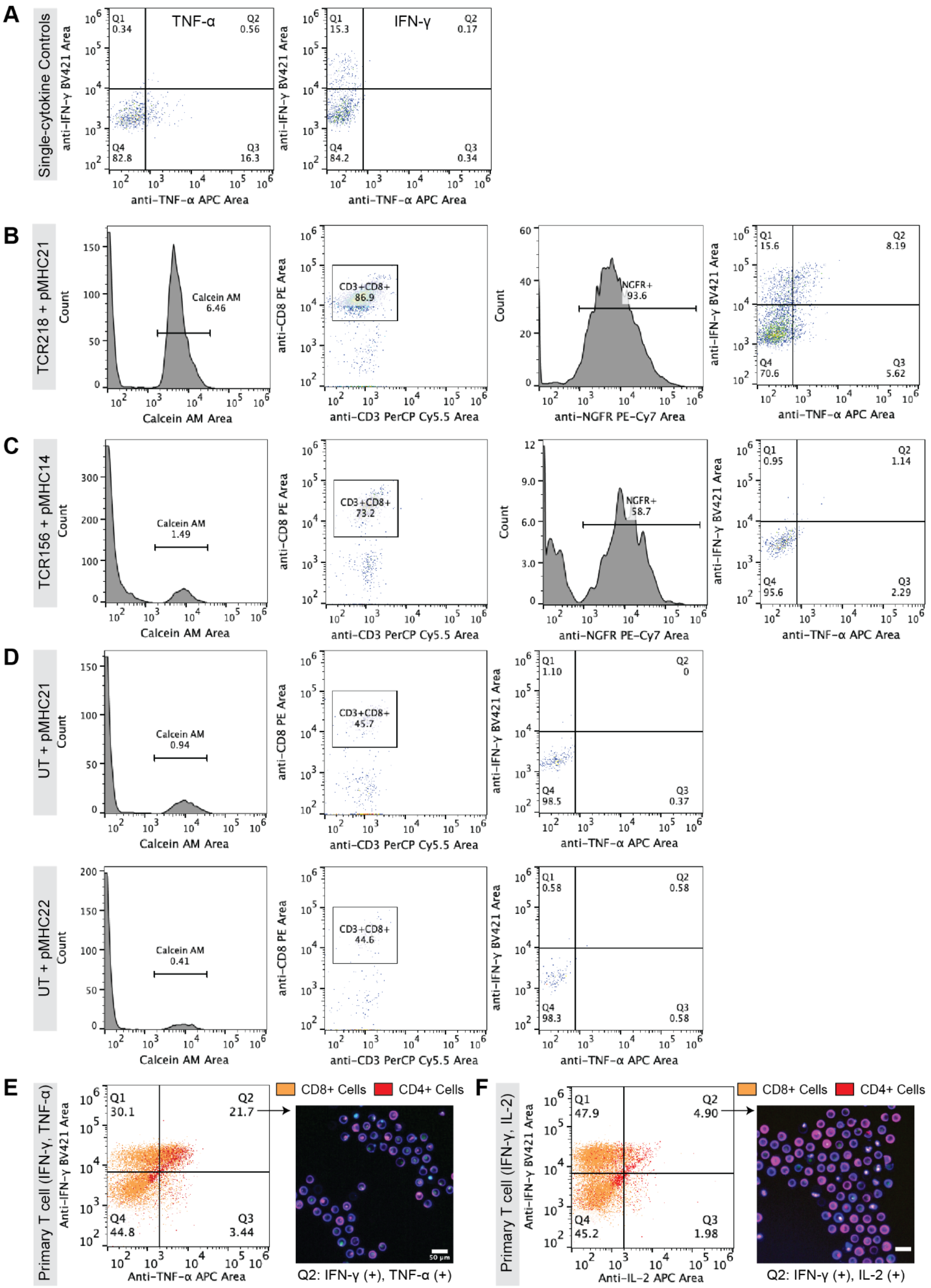
A
Single-cytokine Controls
TNF-α
IFN-γ
anti-IFN-γ BV421 Area
anti-TNF-α APC Area
Q1 0.34
Q2 0.56
Q3 16.3
Q4 82.8
Q1 15.3
Q2 0.17
Q3 0.34
Q4 84.2
B
TCR218 + pMHC21
Count
Calcein AM Area
Calcein AM 6.46
anti-CD8 PE Area
anti-CD3 PerCP Cy5.5 Area
CD3+CD8+ 86.9
anti-NGFR PE-Cy7 Area
NGFR+ 93.6
Q1 15.6
Q2 8.19
Q3 5.62
Q4 70.6
C
TCR156 + pMHC14
Calcein AM 1.49
CD3+CD8+ 73.2
NGFR+ 58.7
Q1 0.95
Q2 1.14
Q3 2.29
Q4 95.6
D
UT + pMHC21
Calcein AM 0.94
CD3+CD8+ 45.7
Q1 1.10
Q2 0
Q3 0.37
Q4 98.5
UT + pMHC22
Calcein AM 0.41
CD3+CD8+ 44.6
Q1 0.58
Q2 0.58
Q3 0.58
Q4 98.3
E
Primary T cell (IFN-γ, TNF-α)
Anti-IFN-γ BV421 Area
Anti-TNF-α APC Area
Q1 30.1
Q2 21.7
Q3 3.44
Q4 44.8
CD8+ Cells
CD4+ Cells
50 μm
Q2: IFN-γ (+), TNF-α (+)
F
Primary T cell (IFN-γ, IL-2)
Anti-IL-2 APC Area
Q1 47.9
Q2 4.90
Q3 1.98
Q4 45.2
Q2: IFN-γ (+), IL-2 (+)

Supplementary Figure 9: Detailed FACS analysis and gating strategy for multiplexed secretion-based profiling using nanovials. (A) Flow cytometry dot plots with gating defining populations positive for IFN-γ and TNF-α secretion on nanovials with single cytokine capture antibodies. (B) Flow cytometry plots for identifying functional antigen-specific T cells transduced with TCR218 and TCR156 loaded on HLA-A*02:01 restricted PAP21 and PAP22 pMHC labeled nanovials, respectively. IFN-γ and TNF-α secretion signals were analyzed from the calcein AM stained, CD3 and CD8 double positive population with NGFR signal above background. (C) Control flow cytometry plots for analyzing secretions from TCR156 transduced PBMCs loaded on non-cognate PAP14 pMHC labeled nanovials or from (D) untransduced PBMCs loaded on PAP21 and PAP22 pMHC labeled nanovials. (E) Multiplexed secretion profiling of untransduced human primary T cells activated by PMA/Ionomycin. Experimental dot plots showing IFN-γ and TNF-α or IFN-γ and IL-2 secretion from activated CD4+ and CD8+ T cells loaded on nanovials. Single cells were sorted based on CD4+ or CD8+ gates as well as the four quadrant gates. Scale bars represent 50 μm.

Clonotype ID	TCR_ID	TRAV	TRAJ	CDR3A	TRBV	TRBD	TRBJ	CDR3B	Frequency	Epitope
clonotype0_2	TCR1	TRAV24	TRAJ21	CAFISFNKFYF	TRBV6-5		TRBJ1-2	CASSPQTGTGTYGYTF	801	CMV1
clonotype0_1	TCR4	TRAV24	TRAJ21	CAFISFNKFYF	TRBV6-5	TRBD1	TRBJ1-2	CASSAQTGAAYGYTF	801	CMV1
clonotype1_1	TCR2	TRAV24	TRAJ49	CARNTGNQFYF	TRBV6-5		TRBJ1-2	CASSAQTGAAYGYTF	308	CMV1
clonotype1_3	TCR3	TRAV24	TRAJ49	CILRDIPFDRGSTLGRLYF	TRBV6-5		TRBJ1-2	CASSAQTGAAYGYTF	308	CMV1
clonotype1_2	TCR5	TRAV26-2	TRAJ49	CARNTGNQFYF	TRBV6-5	TRBD1	TRBJ1-2	CASSPQTGTGTYGYTF	308	CMV1
clonotype1_4	TCR6	TRAV26-2	TRAJ49	CILRDIPFDRGSTLGRLYF	TRBV6-5	TRBD1	TRBJ1-2	CASSPQTGTGTYGYTF	308	CMV1
clonotype4	TCR8	TRAV3	TRAJ31	CAVRDLSARLMF	TRBV12-4		TRBJ2-7	CASSSVNEQYF	41	CMV1
clonotype5	TCR9	TRAV17	TRAJ52	CAMWTSYGKLTF	TRBV7-3		TRBJ2-2	CASSLEVTGELFF	35	CMV1
clonotype11_1	TCR12	TRAV5	TRAJ36	CAERIQTGANNLFF	TRBV13		TRBJ1-1	CASSLGGGGVTEAFF	14	CMV1
clonotype11_3	TCR13	TRAV13-2	TRAJ8	CAERIQTGANNLFF	TRBV24-1		TRBJ1-1	CATTYGRMNTEAFF	14	CMV1
clonotype11_2	TCR14	TRAV5	TRAJ36	CAVFNTGFQKLVF	TRBV13		TRBJ1-1	CASSLGGGGVTEAFF	14	CMV1
clonotype11_4	TCR15	TRAV13-2	TRAJ8	CAVFNTGFQKLVF	TRBV24-1		TRBJ1-1	CATTYGRMNTEAFF	14	CMV1
clonotype9	TCR17	TRAV24	TRAJ49	CALGYGNQFYF	TRBV27	TRBD1	TRBJ1-2	CASSLLATGGNGYTF	10	CMV1
clonotype12_1	TCR21	TRAV24	TRAJ49	CARNTGNQFYF	TRBV6-5	TRBD1	TRBJ1-2	CASSPQTGTGTYGYTF	7	CMV1
clonotype12_2	TCR22	TRAV24	TRAJ49	CARNTGNQFYF	TRBV6-5		TRBJ1-2	CASSRQTGSIYGYTF	7	CMV1
clonotype14_1	TCR23	TRAV3	TRAJ31	CAVRDISARLMF	TRBV12-4		TRBJ1-1	CASSSVTEAFF	6	CMV1
clonotype14_2	TCR24	TRAV10	TRAJ45	CVVIVMYSGGGADGLTF	TRBV12-4		TRBJ1-1	CASSSVTEAFF	6	CMV1
clonotype20	TCR25	TRAV8-3	TRAJ43	CAVAPSNDMRF	TRBV27		TRBJ2-1	CASSLVSGATYNEQFF	5	CMV1
clonotype26	TCR34	TRAV5	TRAJ42	CAEIPNYGGSQGNLIF	TRBV12-4		TRBJ1-2	CASSLVGGRYGYTF	2	CMV1
clonotype51	TCR35	TRAV21	TRAJ49	CAANTGNQFYF	TRBV7-8		TRBJ2-3	CASSLMFTGVPQDTQYF	2	CMV1
clonotype36	TCR38	TRAV24	TRAJ49	CARNTGNQFYF	TRBV6-5		TRBJ1-2	CASSPTTGTAYGYTF	2	CMV1
clonotype3	TCR7	TRAV5	TRAJ37	CAESIGKLIF	TRBV29-1	TRBD1	TRBJ1-4	CSVGHGGTNEKLFF	49	EBV
clonotype6_1	TCR10	TRAV5	TRAJ3	CAEYSSASKIIF	TRBV14		TRBJ2-1	CASSQSPGGTQFF	26	EBV
clonotype6_2	TCR11	TRAV4	TRAJ16	CLVGDGKSDGQKLLF	TRBV14		TRBJ2-1	CASSQSPGGTQFF	26	EBV
clonotype7	TCR16	TRAV5	TRAJ23	CAESIGKLIF	TRBV29-1	TRBD1	TRBJ1-4	CSVGQGGTNEKLFF	10	EBV
clonotype13_1	TCR18	TRAV5	TRAJ31	CAEDNNARLMF	TRBV20-1	TRBD1	TRBJ1-2	CSARDGTGNGYTF	8	EBV
clonotype13_2	TCR19	TRAV13-1	TRAJ26	CAVYGGQNFVF	TRBV20-1	TRBD1	TRBJ1-2	CSARDGTGNGYTF	8	EBV
clonotype10	TCR20	TRAV5	TRAJ3	CAEYSSASKIIF	TRBV14		TRBJ2-3	CASSQSPGGTQYF	7	EBV
clonotype22	TCR33	TRAV5	TRAJ31	CAEDSNARLMF	TRBV20-1	TRBD1	TRBJ1-2	CSARDGTGNGYTF	4	EBV
clonotype18	TCR32	TRAV3	TRAJ12	CAVRDTRDSSYKLIF	TRBV10-3		TRBJ2-7	CAISEDITVAPEQYF	3	EBV
clonotype45	TCR36	TRAV1-2	TRAJ10	CAVDILTGGGNKLTF	TRBV27		TRBJ1-5	CASGPYEGNQPQHF	2	EBV
clonotype35	TCR37	TRAV29	TRAJ42	CAAGGSQGNLIF	TRBV27		TRBJ1-2	CASSLTGTFRGYTF	2	EBV

Supplementary Figure 10: Detailed list of clonotypes recovered from nanovials (frequency >=2) with corresponding V(D)J αβ genes, CDR3 αβ sequences, frequency of clonotype and epitope information. Sequences colored in pink represent those found in previously reported studies.

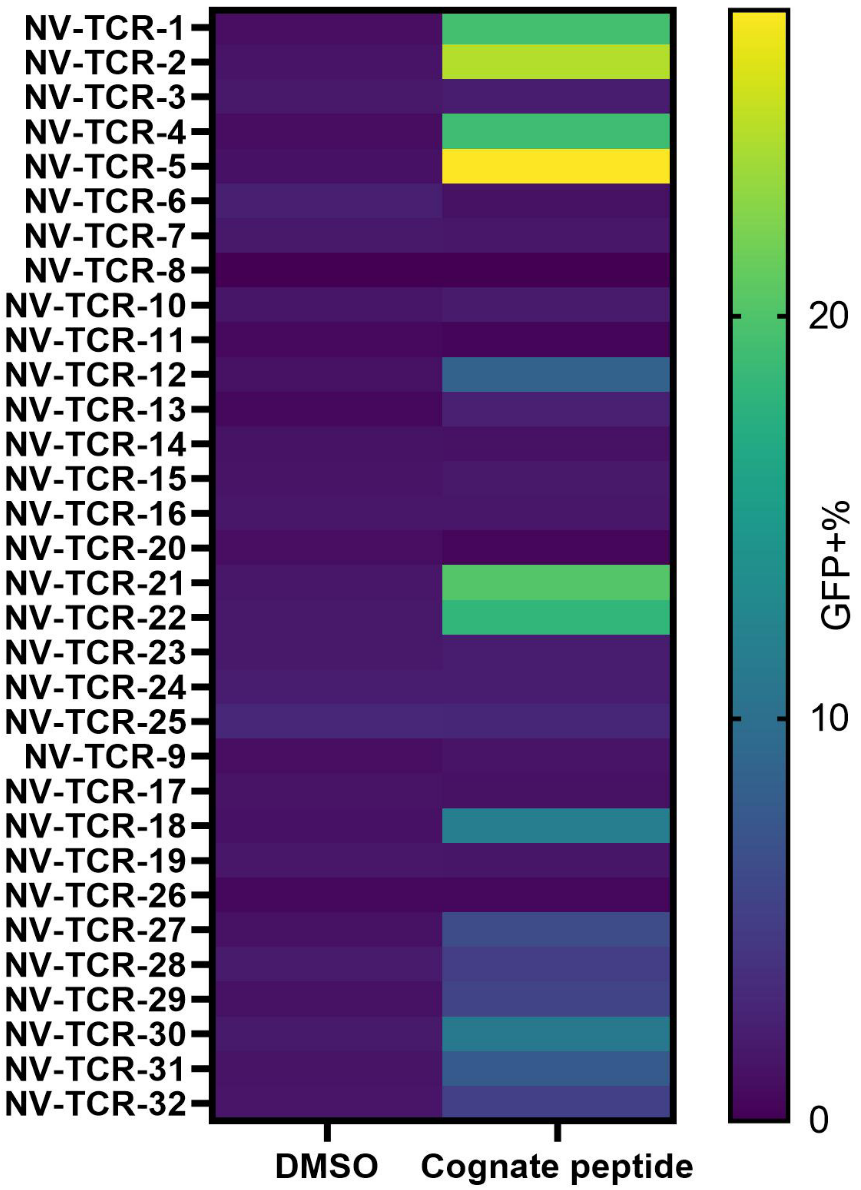

Supplementary Figure 11. Heatmap showing GFP positivity when each TCR was transduced into NFAT GFP reporter cells and were co-cultured with K562 cells presenting exogenously added peptide. K562 cells with DMSO (instead of cognate peptide) were used as a control to measure non-specific activation associated with each TCR.

Supplementary Note

From fluorescence microscopy images, we observed two distinct fluorescence patterns, fluorescence spread across the nanovial cavity, presumably from secreted cytokines and fluorescence associated with cells on nanovials (without signal on the nanovial). We developed an approach to use the fluorescence peak shape to distinguish between nanovial and non-specific cell staining. From fluorescence images of T cells secreting on nanovials, we plotted the fluorescence intensity profile across the cavity diameter using MATLAB and calculated the maximum intensity (height), the area under the intensity curve (area), and the ratio between the area and height (fig. SN.B). Nanovials with both spatially spread secretion signal on their cavities and localized labels bound to the surfaces of adhered cells were found to have a similar range of fluorescence peak height values. However, the area over height measurement was distinctly higher for the nanovials with secretion signal. This information may be used as a distinguishing feature in flow cytometry, as analogous fluorescent pulses are generated when nanovials pass through the excitation laser beam spot (fig. SN.A). The height of the flow cytometry pulse is determined by the maximum fluorescence intensity of the nanovial and the area integrates the intensity emitted over the entire transit event through the laser spot. Accordingly, our nanovials with spatially-distributed secretion signals are expected to produce higher fluorescence area signals for a given fluorescence intensity (height) compared to nanovials with cells bound to labels. We analyzed our samples based on a combination of fluorescence peak area and peak height signals (area vs. height plot) and observed two populations, where one population had higher area signal as compared to the other population with similar height values. When we sorted nanovials with larger ratios of area/height (2.06% high secretion and 2.77% low secretion gates), the recovered nanovials had higher secretion signals, while sorted events in the lower area/height (A/H) region (2.61% label binding to cell gate) corresponded to nanovials with label bound to cells (fig. SN.C). Using this area vs. height metric, we were able to sort populations of cells with secreted cytokine signal only (A/H > 3), completely differentiating secretion signal on nanovials from signal solely from cell surface binding or intracellular staining of permeabilized or dead cells (fig. SN.D). The percent of the cell population with label bound was consistent across all three cytokines (~2.6% of the total analyzed events).

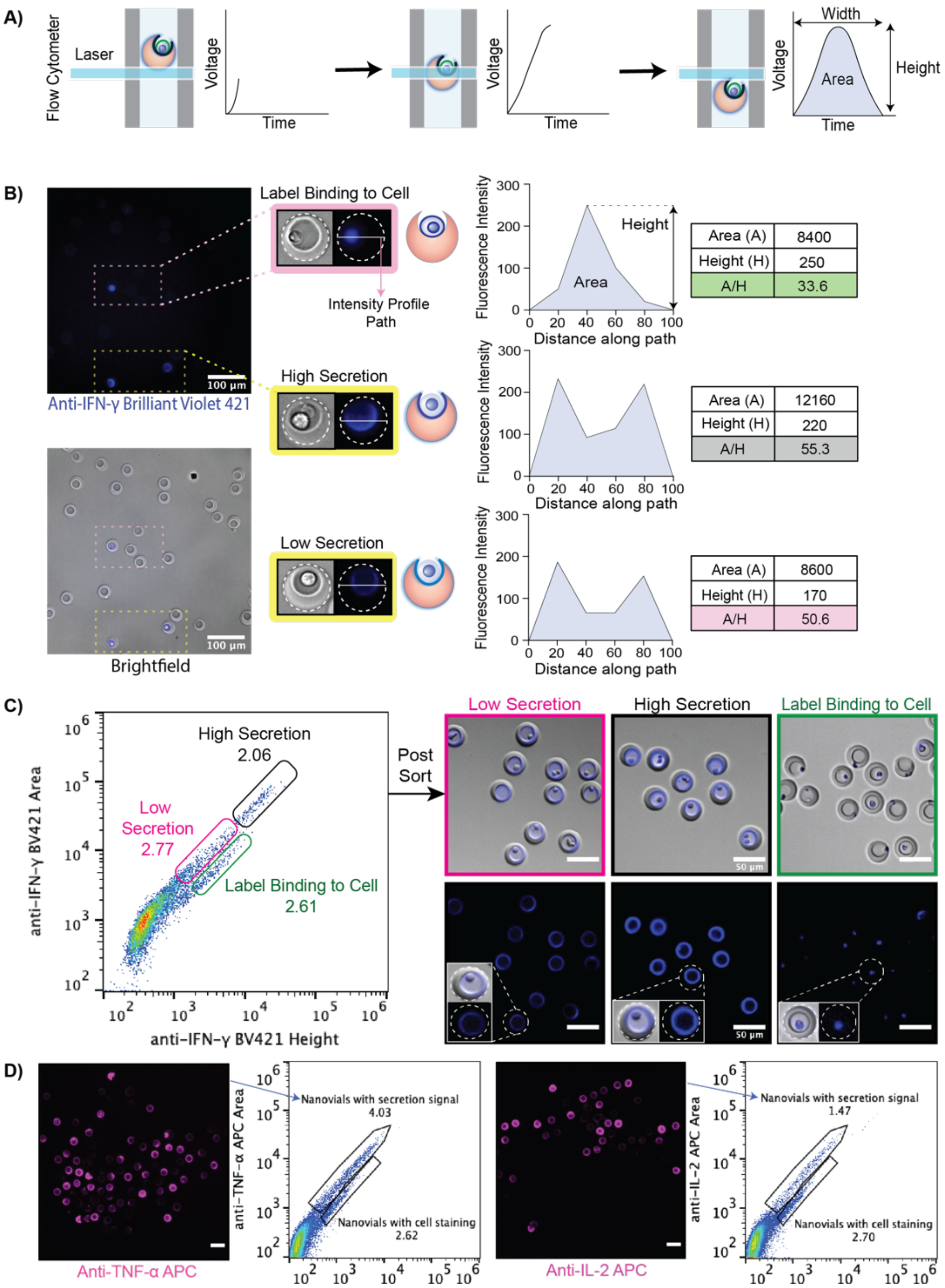

Supplementary Note Figure 1: Using the fluorescence peak area and height to measure single-cell secretions on nanovials. A) Overview schematic of the nanovial fluorescence peak shape obtained when a nanovial transits a laser spot in a flow cytometer. B) Fluorescence microscopy images of pre-sort nanovials with cells showing two distinct fluorescence patterns, with dotted lines in insets outlining the nanovial boundaries: fluorescence spread across the nanovial cavity from secreted cytokines or fluorescence associated with cells on nanovials without signal across the cavity area. The fluorescence intensity profile was computed across the cavity of each image and the maximum intensity (height, H), area under the intensity curve (area, A), and the ratio between the area and height were calculated. Scale bars represent 100 μm. C) Isolation of T cells on nanovials with secretion signal. Three different gates were used to differentiate spatially-extended IFN-γ secretion signals on nanovials (pink and black secretion gates) from signal solely from non-specific cell surface binding (green label binding to cell gate). Sorted cells on nanovials have different distribution of fluorescence signal, as shown in the images. Scale bars represent

50 μm. D) Nanovials with each cytokine (TNF-α or IL-2) secretion signal were sorted using area vs. height metrics. The ability to isolate on-nanovial cytokine staining was consistent across different cytokines as shown in fluorescence microscopy images. Scale bars represent 50 μm.

Supplementary Materials and Methods

Nanovial secretion assay general procedure

Cell loading onto nanovials. Each well of a 24-well plate was filled with 1 mL of media and 30 μL of reconstituted functionalized nanovials (6 μL of nanovial volume=187,000 total nanovials) was added in each well using a standard micropipette. Cells were seeded in each well and extra culture medium was added to make a total volume of 1.5 mL. Each well was mixed by simply pipetting 5 times with a 1000 μL pipette set to 1000 μL. The well plate was transferred to an incubator to allow cell binding; the volume in each well was pipetted up and down again 5 times with a 200 μL pipette set to 200 μL at 30-minute intervals. After one hour, nanovials were strained using a 20 μm cell strainer to remove any unbound cells and recovered. During this step, any unbound cells were washed through the strainer and only the nanovials (with or without cells loaded) were recovered into a 12-well plate with 2 mL of media by inverting the strainer and flushing with media.

Activation, secretion accumulation and secondary antibody staining on nanovials. After cell loading, cells on nanovials in a 12-well plate were activated via 10 ng/mL PMA (Sigma) and 2.5 μM ionomycin (Sigma) or the pMHCs on the nanovials for three hours in the incubator. Each sample was recovered in a conical tube with 5 mL wash buffer and centrifuged for 5 minutes at 200xg. Supernatant was removed and nanovials were reconstituted at a ten-fold dilution in Washing Buffer containing detection antibodies to label secreted cytokines and/or cell surface markers. Concentrations of fluorescent antibodies per 187,000 nanovials (~ 6 μL nanovial volume) are listed in Table 1, unless otherwise stated. A typical experiment used 30 μL of nanovials, which were incubated with 5X the volumes of antibodies listed in Table 1 (i.e. 25 μL anti-IFN-γ BV421, 10 μL anti-CD3 PerCP Cy5.5 and 10 μL anti-CD8 PE) at the total reaction volume of 300 μL. Nanovials were incubated with the detection antibody cocktail at 37°C for 30 minutes, protected from light. After washing nanovials with 5 mL of Washing Buffer, nanovials were resuspended at a 50-fold dilution in Washing Buffer and transferred to a flow tube.

Table 1. Secondary antibody concentrations per 6 μL nanovial volume.

Calcein AM	Anti-IFN-γ BV421	Anti-TNF-α APC	Anti-IL-2 APC	Anti-NGFR PE-Cy7	Anti-CD8 PE	Anti-CD3 PerCP Cy5.5	Anti-CD3 APC Cy7	Anti-granzyme B APC
Thermo Fisher C3099	Biolegend 502532	Biolegend 502912	BD Sciences 554567	Biolegend 345110	Invitrogen 1208842	Biolegend 300430	Biolegend 300426	Biolegend 372203
0.3 μM	5 μL of 100 ug/mL	5 μL of 100 μg/mL	5 μL of 200 μg/mL	2 μL of 100 μg/mL	2 μL of 125 μg/mL	2 μL of 100 μg/mL	2 μL of 200 μg/mL	5 μL of 100 μg/mL

Labeling of secretion (granzyme B) using oligonucleotide barcoded secondary antibody. Following addition of fluorescent detection antibody (anti-granzyme B APC), nanovials were washed with 5 mL of Washing Buffer and reconstituted at a ten-fold dilution containing 30 nM of TotalSeq™ C-0987 anti-APC antibody (Biolegend, 408007). Nanovial suspension was incubated at 37°C for 30 minutes. After washing nanovials with 5 mL of Washing Buffer, nanovials were resuspended at a 50-fold dilution in Washing Buffer and transferred to a flow tube for FACS analysis and sorting.

Flow cytometer analysis and sorting. All flow cytometry analysis and sorting were performed using the SONY SH800 cell sorter equipped with a 130 micron sorting chip (SONY Biotechnology). The cytometer was configured with violet (405 nm), blue (488 nm), green (561 nm) and red (640 nm) lasers with 450/50 nm, 525/50 nm, 600/60 nm, 665/30 nm, 720/60 nm and 785/60 nm filters. Standard gain settings for different sensors are indicated in table 2 below and gains were adjusted depending on the fluorophores used. In each analysis, samples were compensated using negative (blank nanovials) and positive controls (1000 ng/mL recombinant cytokine captured nanovials labeled with each fluorescent detection antibody or cells stained with each surface marker). Nanovial samples were diluted to approximately 623 nanovial/μL in Washing Buffer for analysis and sorting. Drop delay was configured using standard calibration workflows and single-cell sorting mode was used for all sorting as was previously determined to achieve the highest purity and recovery (1). A sample pressure of 4 was targeted. The following order of gating strategy was used to identify cells on nanovials with strong secretion signal: 1) nanovial population based on high forward scatter height and side scatter area, 2) calcein AM positive population, 3) cell surface marker positive population (CD3, CD8, CD4 or NGFR), 4) cytokine secretion signal positive population based on fluorescence peak area and height.

Table 2. Common gain settings used for analysis and sorting.

Sensor	FSC	BSC	FL1	FL2	FL3	FL4	FL5	FL6
Gain	1	26%	28%	22%	28%	30%	32%	32%

Dynamic range of cytokine detection on nanovials with a combination of antibodies

Nanovials were labeled with biotinylated antibodies (140 nM anti-CD45 and 140 nM anti-IFN-γ or anti-TNF-α) using the modification steps mentioned above. Each sample of cytokine capture antibody-labeled nanovials was incubated with 0, 10, 100, or 1000 ng/mL of recombinant human IFN-γ (R&D Systems, 285IF100) and TNF-α (R&D Systems, 210TA020) for 2 hours at 37°C. Excess proteins were removed by washing nanovials three times with Washing Buffer. Nanovials were pelleted at the last wash step and incubated with anti-IFN-γ BV421 and anti-TNF-α APC as described in secondary antibody staining procedure and Table 1. Following washing three times, nanovials were reconstituted at a 50 times dilution in the Washing Buffer and transferred to a flow tube. Fluorescent signal on nanovials was analyzed using a cell sorter with sensors and gains mentioned in the flow cytometer analysis and sorting section.

Maximum binding of pMHC on nanovials

Streptavidin coated nanovials were functionalized with biotinylated HLA-A*02:01 restricted NY-ESO-1 pMHC by incubating at various concentrations (0, 20, 40, 80, 90, 100 μg/mL) and washed three times as described in Nanovial Functionalization section. Nanovials were reconstituted at a ten-fold dilution in Washing Buffer containing 2 μL of 100 μg/mL anti-HLA-A2 FITC antibody (Biolegend, 343304) and incubated for 30 minutes at 37°C. After washing three times with Washing Buffer, mean fluorescence intensity was measured by flow cytometry.

Single-T cell loading and statistics

Nanovials labeled with anti-CD45 antibodies were prepared using the procedures described above. To test cell concentration dependent loading of nanovials 0.15 x 10^6 (0.8 cells per nanovial), 0.3 x 10^6 (1.6 cells per nanovial), and 0.47 x 10^6 (2.4 cells per nanovial) of cell tracker deep red stained human primary T cells were each seeded onto 187,000 nanovials in a 24-well plate and recovered as described above. Loading efficiency was analyzed using a custom image analysis algorithm in MATLAB. The software measured the total number of nanovials in each image frame, then the number of cells in each nanovial was manually counted to record the total number of nanovials with 0, 1 or 2 or more cells (n > 2000). For comparing loading with different cell binding motifs, nanovials were labeled with 140 nM of each biotinylated antibody: anti-CD3 (Biolegend, 317320), anti-CD3 and anti-CD28 (Biolegend, 302904), or anti-CD45. Nanovials were seeded with 0.3 million cells in each well. To determine the effect of increased anti-CD45 concentration on nanovials, nanovials were labeled by incubating with 0, 70, 140, or 210 nM of anti-CD45 antibodies and seeded with 0.3 million cells in a 24-well plate. After cell binding and recovery of nanovials, the number of cells in each nanovial was analyzed using the same image analysis algorithms mentioned above (n > 2000).

Analysis and sorting of human primary T cells based on secretion level

Nanovials were sequentially coated with streptavidin as described above and incubated with a solution of biotinylated antibodies (140 nM anti-CD45 and anti-IFN-γ, anti-TNF-α or anti-IL-2). 0.3 million human primary T cells were seeded on nanovials as described above and recovered into a 12-well plate in 2 mL of T cell expansion medium with PMA and ionomycin, followed by 3 hours of activation. Secreted cytokines (IFN-γ, TNF-α, IL-2) were labeled with fluorescent detection antibodies at concentrations described in Table 1 and cells were stained with calcein AM viability dye. After resuspending nanovials at 50-fold dilution in Washing Buffer, a small fraction of sample was transferred to a 96-well plate to be imaged using a fluorescence microscope prior to sorting. Pre-sort images were analyzed by custom image analysis algorithms in MATLAB. Fluorescence intensity profiles were calculated along a line segment manually defined around the cavity of nanovial. The intensity peak height and the area under the intensity profile were then evaluated to find the peak area over height aspect ratio. Samples were analyzed using a cell sorter based on a combination of fluorescence area and height signals. To sort live single cells based on secretion signal, nanovials with calcein AM staining were first gated and high, medium, or low secretors were sorted by thresholding the fluorescence area and height signals. Sorted samples were imaged with a fluorescence microscope to validate the enrichment of nanovials based on the amount of secreted cytokine captured on the nanovials.

Capture, activation, and expansion of antigen-specific T cells on pMHC-labeled nanovials

To determine the effect of pMHC concentration on antigen-specific T cell capture efficiency, streptavidin-coated nanovials were functionalized by incubation with different concentrations of biotinylated HLA-A*02:01 NY-ESO-1 pMHCs (10, 20, 40, 80 µg/mL) and seeded with 0.3 million 1G4 TCR transduced PBMCs or untransduced PBMCs. After straining and recovery, samples were stained with calcein AM and anti-NGFR PE Cy7 antibody as described above. The fractions of nanovials with live cells and NGFR positive cells were measured by a cell sorter. To test if activation was specific to the presence of pMHCs on nanovials, 1G4 transduced PBMCs were

loaded onto anti-IFN-γ antibody and pMHC or anti-CD45 labeled nanovials. Following 3 hours of activation, nanovial samples were stained with anti-IFN-γ BV421 and anti-NGFR PE Cy7 antibodies. The fraction of nanovials with NGFR positive cells and secretion signal was identified using flow cytometry. Secretion signal from 1G4 PBMCs on pMHC labeled nanovials was measured at 0, 3, 6, and 12 hour time points. For detachment and expansion of antigen-specific T cells post-sort, 1G4 PBMCs loaded onto pMHC nanovials were sorted based on calcein AM and NGFR signal and reconstituted with 0.75 mL media and 0.25 mL of 10 mg/mL Collagenase Type II solution (STEMCELL Technologies), followed by a 2 hour incubation at 37°C. Samples were vortexed 3 times at 20 second intervals and strained through a 20 μm strainer to remove empty nanovials. Cells cultured for 5 days were stained with 0.3 μM calcein AM and 0.02 mg/mL of propidium iodie or fluorescent anti-NGFR antibody and imaged using fluorescence microscopy or analyzed using flow cytometry for NGFR expression.

Recovery of NY-ESO-1 TCR-transduced cells of various affinities

PBMCs were transduced with five different TCRs (1G4, 3A1, 4D2, 5G6, 9D2). 1 million of each TCR transduced or untransduced cells were seeded with HLA-A*02:01 NY-ESO-1 pMHC and anti-IFN-γ labeled nanovials and activated for 3 hours. Following straining of any unbound cells, recovered samples were stained with a cocktail of detection antibodies (calcein AM, anti-CD3 PerCP Cy5.5, anti-CD8 PE, anti-NGFR PE Cy7, anti-IFN-γ BV421) at concentrations described in Table 1. In parallel, 0.2 million PBMCs transduced with each TCR were stained with dual-color commercial HLA-A*02:01 NY-ESO-1 tetramers (MBL International, TB-M105-1 and TB-M105-2), anti-CD3 PerCP Cy5.5, and anti-CD8 PE antibodies as previously reported (2). Using flow cytometric analysis, the purity of nanovial sample was calculated as the fraction of NGFR+ population from calcein AM+CD3+CD8+ cells on nanovials or the fraction of NGFR+ population from calcein AM+CD3+CD8+ cells with IFN-γ secretion signal. The purity of the tetramer stained samples was calculated as the fraction of NGFR+ population from CD3+CD8+ cells with dual-color tetramer signal. For example, a detailed calculation for the purity of recovered 4A2 TCR transduced PBMCs is shown in Table 3.

Table 3. Calculation for the purity of recovered 4A2 TCR-specific T cells

Tetramer		Nanovials	
CD3+CD8+Tetramer+ Cells	38	CD3+CD8+ Cells<br>CD3+CD8+IFN-γ+ Cells	2537<br>235
CD3+CD8+Tetramer+NGFR+ Cells	15	CD3+CD8+NGFR+ Cells<br>CD3+CD8+IFN-γ+NGFR+ Cells	1996<br>217
Purity of recovered sample	40%	Purity of recovered sample	79% (due to binding alone)<br>92% (with secretion signal)

Recovery of TCRs using single-cell TCR αβ sequencing

The standard protocol for 10X Chromium single cell 5' and V(D)J enrichment with feature barcodes was followed unless otherwise noted. Sorted samples reconstituted at 18 μL were loaded into the 10X Chromium Next GEM Chip K for partitioning each nanovial or cell into droplets containing primers specific for the constant region of the V(D)J locus allowing the PCR amplification and enrichment of matched α and β TCR sequences for individual cell barcoded cDNA. Single-cell TCR V(D)J and feature barcode libraries were constructed using the

manufacturer-recommended protocol by the UCLA Technology Center for Genomics & Bioinformatics. Libraries were then sequenced on NextSeq 500 Mid Output with 2x150 bp (Illumina). The Cell Ranger VDJ pipeline was used for sample de-multiplexing and barcode processing.

For recovery of prostate cancer epitope-specific T cells specifically, 10X Chromium single cell 5' GEX and V(D)J enrichment with feature barcode system was utilized. Single-cell TCR V(D)J, 1st feature barcode for specific pMHC molecule (of 10 types) on nanovial, 2nd feature barcode for granzyme B secretion level (oligo-anti-APC expression), and gene expression libraries were constructed using the manufacturer-recommended protocol. Libraries were then sequenced on NextSeq500. The Cell Ranger V(D)J pipeline was used for sample de-multiplexing and barcode processing. Gene expression data set was also analyzed using Cell Ranger Multi v6.1.2 pipeline with Human (GRCh38) 2020-A and Human (GRCh38) v5.0.0 references.

Supplementary References

1. J. de Rutte, R. Dimatteo, S. Zhu, M. M. Archang, D. di Carlo, Sorting single-cell microcarriers using commercial flow cytometers. *SLAS Technol* (2021) https:/doi.org/10.1016/J.SLAST.2021.10.004 (April 9, 2022).
2. P. A. Nesterenko, *et al.*, Droplet-based mRNA sequencing of fixed and permeabilized cells by CLInt-seq allows for antigen-specific TCR cloning. *Proc Natl Acad Sci U S A* **118**, e2021190118 (2021).
